# Allogeneic stem cell strategies in bone regeneration: A systematic review and meta-analysis

**DOI:** 10.1177/20417314261488564

**Published:** 2026-09-17

**Authors:** Paul Åsmund Vågen, Lado Lako Loro, Samih Mohamed-Ahmed, Stein Atle Lie, Mohammed Ahmed Yassin, Cecilie Gjerde

**Affiliations:** 1 Center of Translational Oral Research (TOR), Tissue Engineering Group, Department of Clinical Dentistry, 1658 University of Bergen, Bergen, Norway; 2 Section for Oral and Maxillofacial Surgery, 74812 Ålesund Hospital, Møre & Romsdal Health Trust, Ålesund, Norway

**Keywords:** allogeneic MSC, immune response, bone regeneration, bone defect, biomaterials

## Abstract

Autologous mesenchymal stem cells (MSC) are widely regarded as the gold standard for stem cell-based bone regenerative applications due to their strong osteogenic potential. This systematic review with meta-analysis aims to evaluate existing evidence on the use of allogeneic MSC as an alternative, comparing their regenerative efficacy, immunological response, and clinical outcomes to autologous MSC, including their performance when combined with various biomaterial scaffolds. Based on the objective requirements and inclusion criteria, an initial search was conducted in two databases, Medline and Embase. Review articles were excluded, leaving only individual studies. These were manually evaluated, first by assessing the title and abstract before conducting a full review of the remaining articles. Compliance with the ARRIVE guidelines was evaluated for quality assessment. Thirteen studies met the inclusion criteria, comprising various animal models and one human study. Overall, bone regeneration outcomes were comparable between autologous and allogeneic MSC. Quantitative analyses (micro-computed tomography (µCT), radiography, histology) showed similar bone volume and mineralization across groups, with some studies reporting slightly higher values for autologous MSC. The meta-analysis of nine studies revealed a small effect favoring autologous MSC, however not statistically significant (Hedges’ g = 0.26; 95% CI: 0.04–0.56), with low heterogeneity (I^2^ = 11.4%). Allogeneic MSC induced only transient or mild immune responses without impairing bone healing. Findings from the included human trial supported preclinical evidence, indicating that allogeneic MSC are safe and feasible for clinical application. The potential for using allogeneic instead of autologous MSC appears promising. Overall, the literature indicates no substantial differences between the use of allogeneic and autologous MSC. Lack of standardization of data underscores the need for further research. Additional human studies are needed to validate the results obtained from animal studies.

## 1. Introduction

Bone regeneration is a complex and highly regulated physiological process involving cellular signaling, matrix remodeling, vascularization and immune coordination.^1^ It is critical not only for skeletal development, but also for the repair of fractures and large anomalies.^2^ While minor bone injuries typically heal through endogenous repair mechanisms, large and critical-sized defects often require clinical intervention. Reconstructing critical size bone defects caused by trauma, infectious diseases, congenital abnormalities or tumors remains a significant challenge. Autologous bone grafts have long been regarded as the gold standard in bone regeneration, owing to their unique combination of osteogenic, osteoconductive and osteoinductive properties that collectively support robust and reliable bone healing.^3^ However, autologous grafts are limited by several factors, including donor site morbidity, limited graft quantity, increased surgical time, and unpredictable resorption.^4^ Alternatives like allogeneic bone, xenografts, and alloplastic substitutes have not demonstrated equal regenerative potential.^5^

In recent decades, mesenchymal stem cells (MSC) have gained significant attention as a compelling alternative to conventional bone grafts, particularly within the framework of tissue engineering strategies for bone regeneration.^6^ MSC are multipotent progenitor cells capable of differentiating into various cells including osteoblasts, chondrocytes, and adipocytes under specific conditions.^7^ Their capacity to secrete pro-regenerative cytokines, modulate immune responses, and integrate into host tissues makes them particularly valuable for regenerative medicine.^8^ In the context of bone tissue engineering, MSC have shown the potential to accelerate bone healing, promote angiogenesis, and reduce inflammation, especially when combined with suitable scaffold materials that provide three-dimensional (3D) mechanical support and facilitate cell attachment and growth.^9^

Autologous MSC, typically isolated from bone marrow (BM-MSC) or adipose tissue (AT-MSC), are favored due to their low immunogenicity and reduced risk of graft-versus-host disease.^10^ However, several practical and biological challenges limit their broad application. Harvesting autologous cells often involves invasive procedures, and the yield and potency of these cells may decline with age or in patients with chronic diseases such as diabetes or osteoporosis.^11^ Moreover, autologous MSC may require extensive *in vitro* expansion to reach therapeutic quantities, introducing delays and potential phenotypic drift during cell culture.^12^

To address these limitations, allogeneic MSC derived from healthy donors have gained consideration as a scalable, “off-the-shelf” solution. Allogeneic MSC can be harvested in large quantities, banked under Good Manufacturing Practice (GMP) conditions, and made readily available for clinical use, reducing treatment delays and standardizing therapeutic dosing.^13^ Their immunological behavior is particularly noteworthy: MSC exhibit low expression of MHC class II molecules and co-stimulatory markers (CD80, CD86), enabling them to partially evade immune recognition and suppress host immune responses through the secretion of anti-inflammatory cytokines such as IL-10 and TGF-β.^14,15^

Preclinical studies have demonstrated the capacity of allogeneic MSC to enhance bone formation in both small and large animal models.^16,17^ In many cases, these outcomes were comparable to those achieved with autologous MSC, particularly when allogeneic cells were combined with osteoconductive/osteoinductive scaffolds such as hydroxyapatite, β-tricalcium phosphate, or collagen composites.^18^ Furthermore, some early-phase human trials have suggested that allogeneic MSC can be administered safely, with no significant increase in immune complications or graft failure.^19^ Nonetheless, the clinical application of allogeneic MSC remains contentious, with ongoing concerns regarding their long-term safety, potential immunogenicity, and variability in therapeutic efficacy. Although MSC are relatively immune-evasive, repeated or high-dose administration may provoke adaptive immune responses, particularly if the MSC express donor-specific antigens or differentiate into immunogenic cell types.^20^ Some studies have reported donor-specific antibody formation and T-cell activation following allogeneic MSC transplantation, although the clinical implications of these findings are not fully understood.^21^

Given the rapid advancement of regenerative medicine and the clinical demand for effective bone regeneration therapies, it is essential to conduct a comprehensive evaluation of the current literature. A systematic review and meta-analysis can provide critical insights into the comparative efficacy, immunological safety, and translational viability of allogeneic MSC. Moreover, such an approach can help elucidate key determinants of regenerative outcomes, including the characteristics of biomaterial scaffolds, particularly 3D architectures as well as the choice of experimental models and delivery methodologies. By synthesizing available evidence, we aim to inform future clinical trials, standardization efforts, and regulatory frameworks that will shape the future use of MSC in bone tissue engineering.

The aim of this systematic review and meta-analysis was to comprehensively evaluate and compare the efficacy of autologous versus allogeneic stem cells in bone regeneration. By synthesizing published evidence, the objective is to determine whether allogeneic MSC offers comparable or superior outcomes to autologous MSC in terms of regenerative capacity, clinical feasibility, and immunological safety. Emphasis is placed on their application in reconstructing critical-sized bone defects, such as those in the craniofacial region. Given the advantages of allogeneic cells including greater availability, lower cost, and the absence of donor site morbidity, this study seeks to clarify their potential as a viable and scalable alternative to autologous MSC in clinical bone tissue engineering. By consolidating the available head-to-head evidence, this review seeks to clarify whether allogeneic MSC represents a comparable and clinically viable alternative to autologous MSC, and to identify the key sources of methodological heterogeneity that should inform the design of future confirmatory clinical trials.

## 2. Materials and methods

### 2.1. Search strategy

The search for articles for this systematic review began in April 2024. The database search was conducted using PubMed/Medline and Embase, and relevant peer-reviewed journals written in English were considered. The search strategy combined terms for bone regeneration with terms for allogeneic and autologous stem/stromal cell populations. For bone regeneration, we used text words related to bone and osteogenesis, including *bone*, *bones*, and *osteo* combined with *regenerate*, *regrowth*, *defect*, *repair*, *heal*, *form*, and *osteosis*. For the cell therapy concept, we used text words for both allogeneic and autologous/autogenic/autogeneic cells combined with terms including *ASC*, *MSC*, *BMSC*, *multipotent*, *multi-potent*, *mesenchymal*, *progenitor cell*, *stromal cell*, and *stem cell*. Adjacency operators were applied to capture words appearing within 3 words for the bone regeneration concept and within 5 words for the cell-related concepts. Searches were limited to the title, abstract, and keyword heading word fields. The complete search strategy can be found in supplementary data.

Abstracts translated from other languages to English were also considered. After the initial search in April 2024, an updated search using identical search terms, databases, and eligibility criteria was performed regularly up to 15 March 2026, to ensure the review reflected literature published up to the point of submission. No additional eligible studies were identified. All articles in the search were evaluated by two independent researchers (PÅV and LLL).

#### 2.1.1. Data inclusion criteria

Inclusion criteria required titles and keywords containing combinations of terms such as autologous stem cells, allogeneic stem cells, bone regeneration and bone defects. Only studies that directly compare the use of autologous- and allogeneic stem cells for bone regeneration were considered eligible for inclusion. To ensure comprehensive coverage and minimize the risk of omitting relevant studies, a broad database search strategy was employed.

Eligible animal models included both small (rodent, rabbit) and large (dog, sheep, pig) animal models bearing a surgically created, critical-size or non-spontaneously-healing bone defect, as well as human clinical studies. Studies were required to report a minimum post-implantation follow-up period of 4 weeks to be eligible for inclusion. Eligible outcome measures included at least one quantitative or semi-quantitative assessment of new bone formation, such as radiography, micro-computed tomography (µCT), histomorphometry, or mechanical testing.

#### 2.1.2. Data exclusion criteria

Exclusion criteria included *in vitro* studies only and full text publications in languages other than English. After the initial keywords search, all studies containing allogenic and autologous MSC were reviewed. Studies that did not directly compare both allogeneic and autologous MSC were excluded from the analysis. Studies using genetically modified MSC (e.g., viral transduction, gene knock-in/knock-out) or MSC subjected to *ex vivo* preconditioning protocols (e.g., hypoxic or cytokine preconditioning) prior to implantation were excluded from the systematic review and meta-analysis, given the distinct biological properties conferred by these modifications; such strategies are instead discussed narratively as background in Section 4.4.1.

### 2.2. Study selection

After removing duplicates and cross-checking the articles, two reviewers (PÅV and LLL) independently evaluated all titles and abstracts for inclusion using the criteria described above. Discrepancies between reviewers were resolved through direct discussion, ensuring that only studies aligned with the combined keyword criteria and the overarching aim of the review were included. In the final stage, the full texts of the remaining articles were thoroughly reviewed to determine their compliance with both the inclusion and exclusion criteria.

### 2.3. Data extraction

Relevant data such as population, interventions, comparison and outcomes and study design (PICOS) were extracted from the included studies according to PRISMA guidelines.^22^ *Population*: Animal species or patient characteristics, sample size, and details of the bone defect (location, size, confirmation of critical-sized defect). *Intervention*: Type of stem cells (autologous or allogeneic), cell source, dose and pre-implantation modification and delivery method, with or without biomaterial scaffold. *Comparison*: Opposing stem cell group (autologous vs. allogeneic) and any additional control groups (e.g., empty defect, acellular scaffold). *Outcomes*: Measures of bone regeneration (µCT, histology), biomechanical testing, immunological responses, and reported adverse events. *Study Design*: Study type, randomization/blinding, follow-up duration, and ethical approval. Following the identification of eligible studies, relevant data were systematically extracted and organized into comparative tables (Tables 2–4) to facilitate further analysis and interpretation.

### 2.4. Quality assessment and risk of bias

The methodological quality of the included animal studies was analyzed according to SYRCLE’s risk of bias tool for animal studies.^23^ The answer on the included main 10 questions (tools) should be either with “yes” that indicated low risk of bias, or “no” that indicated high risk of bias. For unclear items an answer with “unclear” was assigned (Supplementary Table).

### 2.5. Meta-analysis

The initial objective of this study was to assess the feasibility of performing a meta-analysis based on quantifiable data indicating the extent of bone volume increase in studies where both autologous and allogeneic MSC were utilized. However, in addition to the variability in measurement techniques employed across studies, few provided the exact numerical values, but some studies included bar charts depicting the relevant measurements. To obtain the underlying data, email requests were sent to the corresponding authors of the articles included. Since none of the authors could provide further information, data was extracted by measuring directly from the graphs

Additional inclusion criteria were monitored, and a checklist was prepared (Table 1) to assess the relevance of the included *in vivo* studies in the meta-analyses study and to minimize data heterogenicity. The quantitative measure of bone regeneration for autologous and allogeneic MSC (effect size) was calculated and compared for each time point across rabbit, rat, dog, sheep, pig, human and mouse models.

**Table 1.** Check lists of the included studies in meta-analysis.

Main check list for included studies in meta-analysis	Additional check list to achieve homogenous data analysis
Direct head-to-head comparison of autologous vs. allogeneic MSC	Excluding added biological factors
*In vivo* animal model	Non *in vitro* studies only
Bone grafting in general with MSC	Excluding partial-thickness defects
Human trials	Excluding modified versions of allogeneic or autologous MSC
Defined study parameters and number of animal (n)/group	
Histomorphometric quantification (from histological sections)	
µCT, histomorphometry	
Biomaterials	

A meta-analysis was conducted in STATA Statistical Software 19 (StataCorp LP, College Station, TX, USA) using a DerSimonian–Laird random-effects model to account for expected heterogeneity across animal species, defect models, scaffolds, and outcome measures. Standardized mean differences (Hedges’ g) were calculated to compare autologous versus allogeneic MSC for quantitative bone-regeneration outcomes (e.g., radiographic/µCT-derived bone formation metrics and histomorphometric new bone measures). When numerical values were not reported, data was extracted from published graphs. Pooled estimates are presented with 95% confidence intervals (CI), and statistical heterogeneity was assessed using the I^2^ statistic, with I^2^ > 75% indicating substantial heterogeneity. If sufficient data was available, subgrouping was performed by defect category and/or anatomical site. We performed separate analyses to see whether differences in study design or treatment characteristics were associated with differences in the observed treatment factors such as species/model, defect location/size, scaffold/carrier type, cell source (BM-MSC vs AT-MSC), osteogenic pre-induction (yes/no), and follow-up time. For studies that did not report exact numerical values in text or tables, quantitative outcome data were obtained by manually measuring the relevant parameters from published figures using a ruler; extraction was performed independently by PÅV and LLL.

## 3. Results

### 3.1 Systematic search outcomes

The detailed screening process is illustrated in Figures 1 and 2. A total of 228 records were identified in the electronic search, which included 98 from Medline and 130 from Embase. Eight articles were duplicates found in both search engines and were initially selected. Based on title and abstract screening, 46 articles were initially selected. After a thorough full text review 33 articles were excluded based on the inclusion and exclusion criteria. 13 studies met the requirements for inclusion.

**Figure 1.**
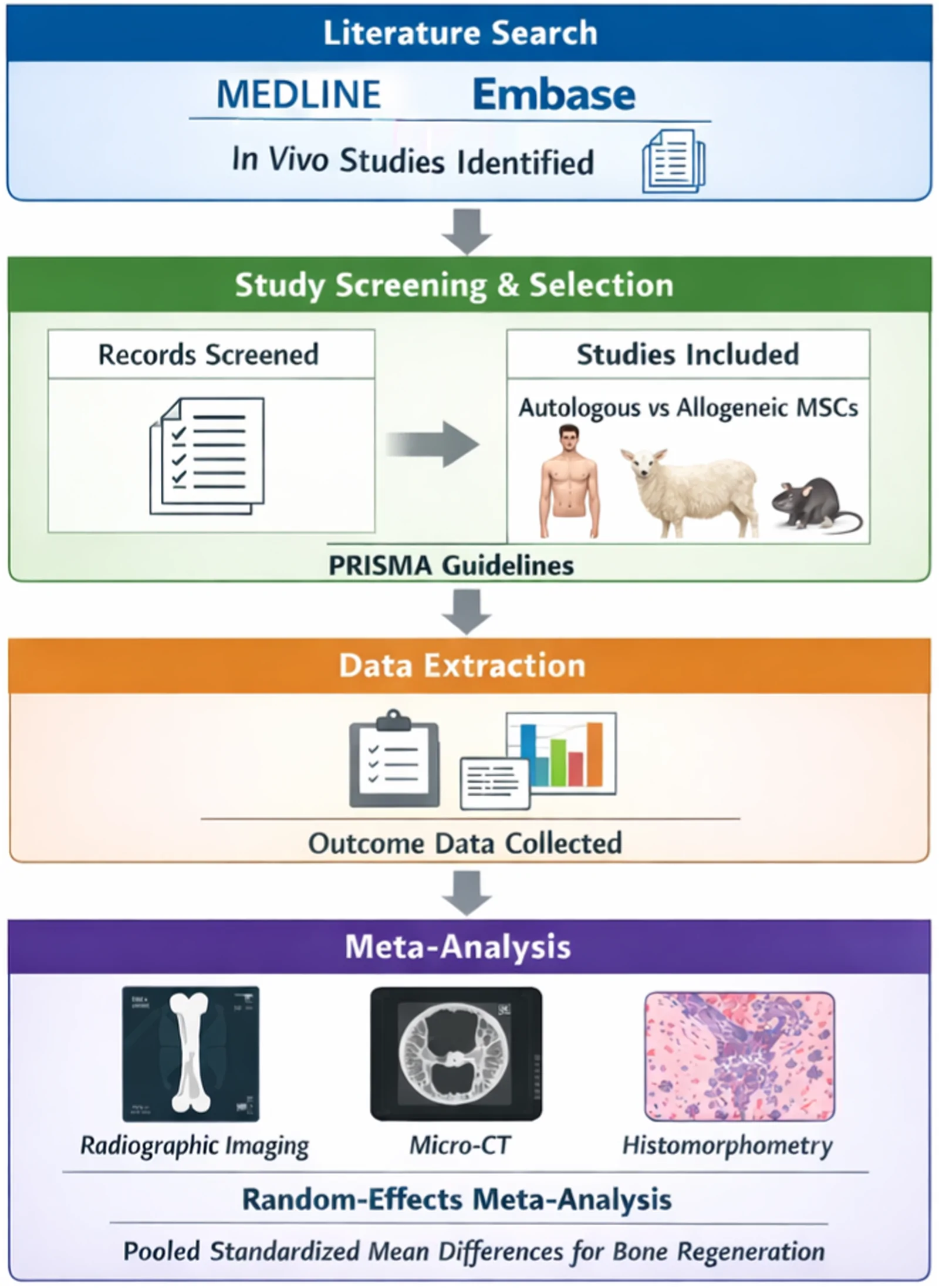
Illustrative overview of findings in the materials and methods section.

**Figure 2.**
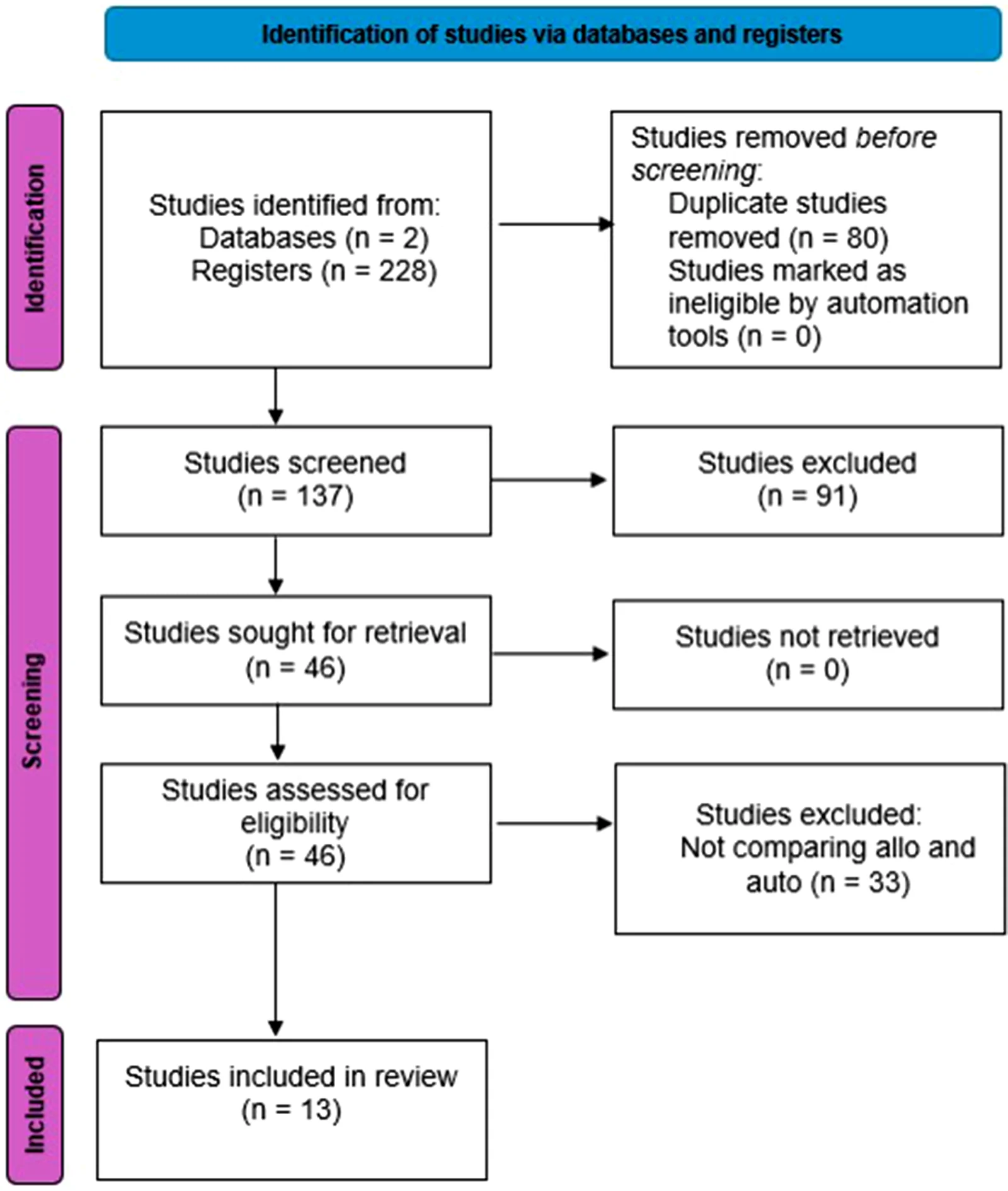
Flowchart for the study screening and selection process and reasons for inclusion/exclusion. n = number of publications. *Studies that were removed for “other reasons” included, among others, being published in a language other than English or the full article not being available.

### 3.2. Variations in methodology and outcome evaluation

The included studies examined various animal species (Table 2 and Figure 3), rats (n=2), rabbits (n=3), dogs (n=4), pigs (n=1), sheep (n=1) and humans (n=1). In addition, one included study used a humanized mouse model to better reflect human immune responses.

**Table 2.** Summary of animal characteristics.

Study	Species (n)	Strain/breed	Age (weeks/months)	Gender	Weight (kg)
1. Arinzeh et al. 2003^24^	Dogs (12)	Coonhound	NA	NA	22-25
2. De kok et al., 2003^25^	Dogs (16)	Beagle	NA	M=F	NA
3. Guo et al., 2009^16^	Mini-pigs (16)	Guizhou Xiang Guangxi Bama	10-12 m	M=F	25-34
4. Kang et al., 2013^26^	Rabbits (19)	New Zealand White	NA	M	2-2,5
5. Liu et al., 2013^27^	Dogs (12)	Beagle	12-18 m	M=F?	22,6-27,2
6. Udehiya et al., 2013^28^	Rabbits	New Zealand White	6-7 m	M & F	1,4-2,2
7. Berner et al., 2013^29^	Sheep (24)	Merino	6-7 y	NA	45±2
8. Wu et al., 2016^30^	Dogs (12)	Beagle	12 m	M	x̄ 16.7
9. Maiti et al., 2018^31^	Rabbits (36)	New Zealand white	7-12 m	M	​
10. Rapp et al., 2018^32^	Humanized mice	NOD/scid IL2Rγc null	6-8 w	MNA	2.78 ± 0.36
11. Pan et al. 2021^17^	Rats (30)	Sprague Dawley	16 w	M	NA
12. Chu et al. 2021^33^	Rats (20)	Lewis, Wistar	8 w	NA	NA
13. Delclós et al., 2024^34^	Humans (12)	​	41.2 y (±12.2)	66.7% M	NA

Age in weeks (wks) and months (m).

NA – not available.

**Figure 3.**
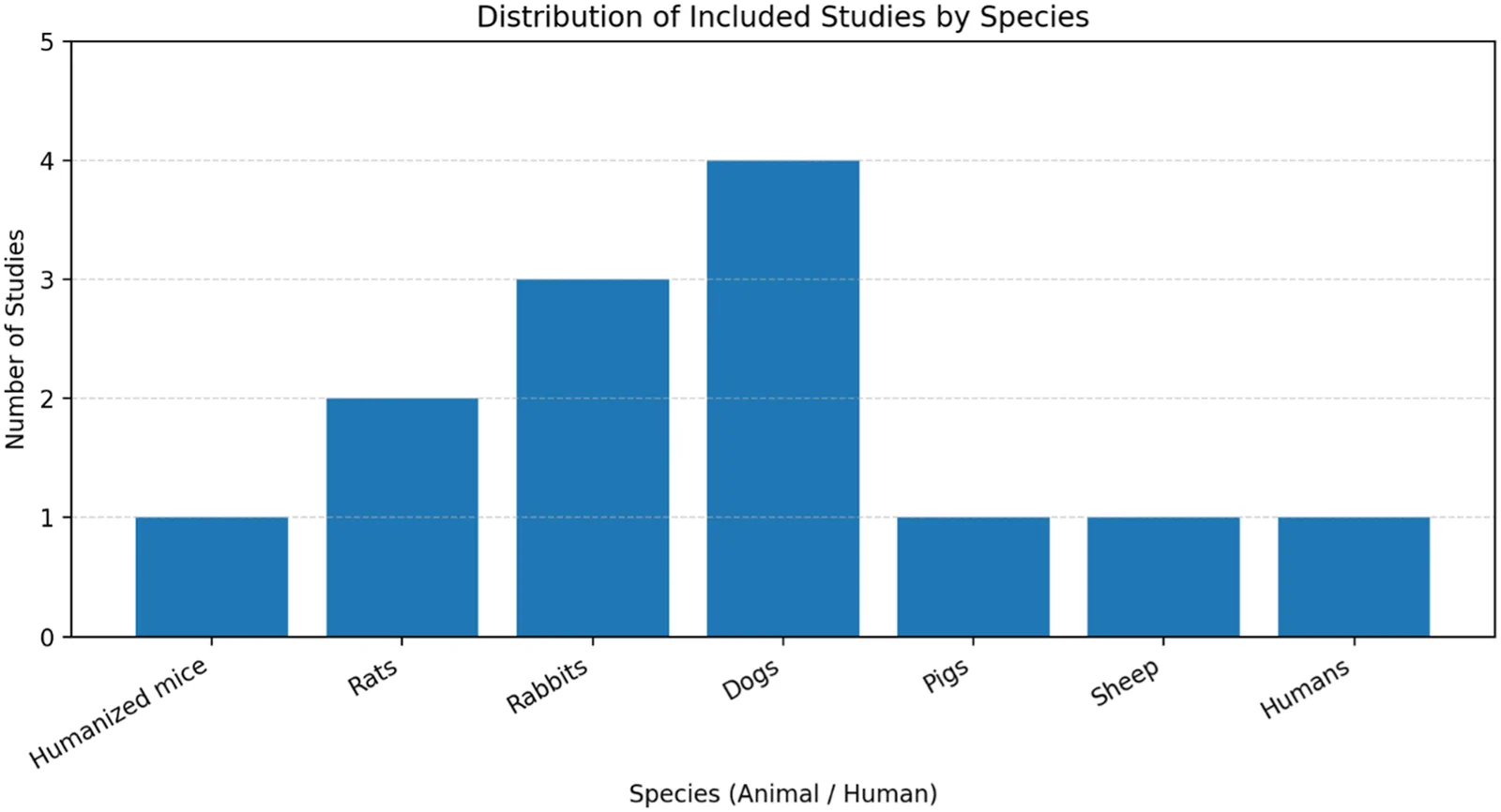
Overview of species represented in the included studies of the systematic review.

Across the 13 included studies, defect sites were distributed across both craniofacial and appendicular long-bone models (Table 2, Figure 4). Based on the study characteristics in Table 3, the most frequent sites were tibia (n=3) and radius (n=3), followed by femur (n=2) and cranium/cranial defects (n=2). Single studies examined mandibular defects (n=1) and alveolar defects (n=1), and the clinical phase II study addressed atrophic non-union in long bones (n=1). When grouped anatomically, 9/13 studies targeted appendicular long bones (tibia, radius, femur, and long-bone non-union), whereas 4/13 studies addressed craniofacial/jaw sites (cranium, mandible, and alveolar bone).

**Figure 4.**
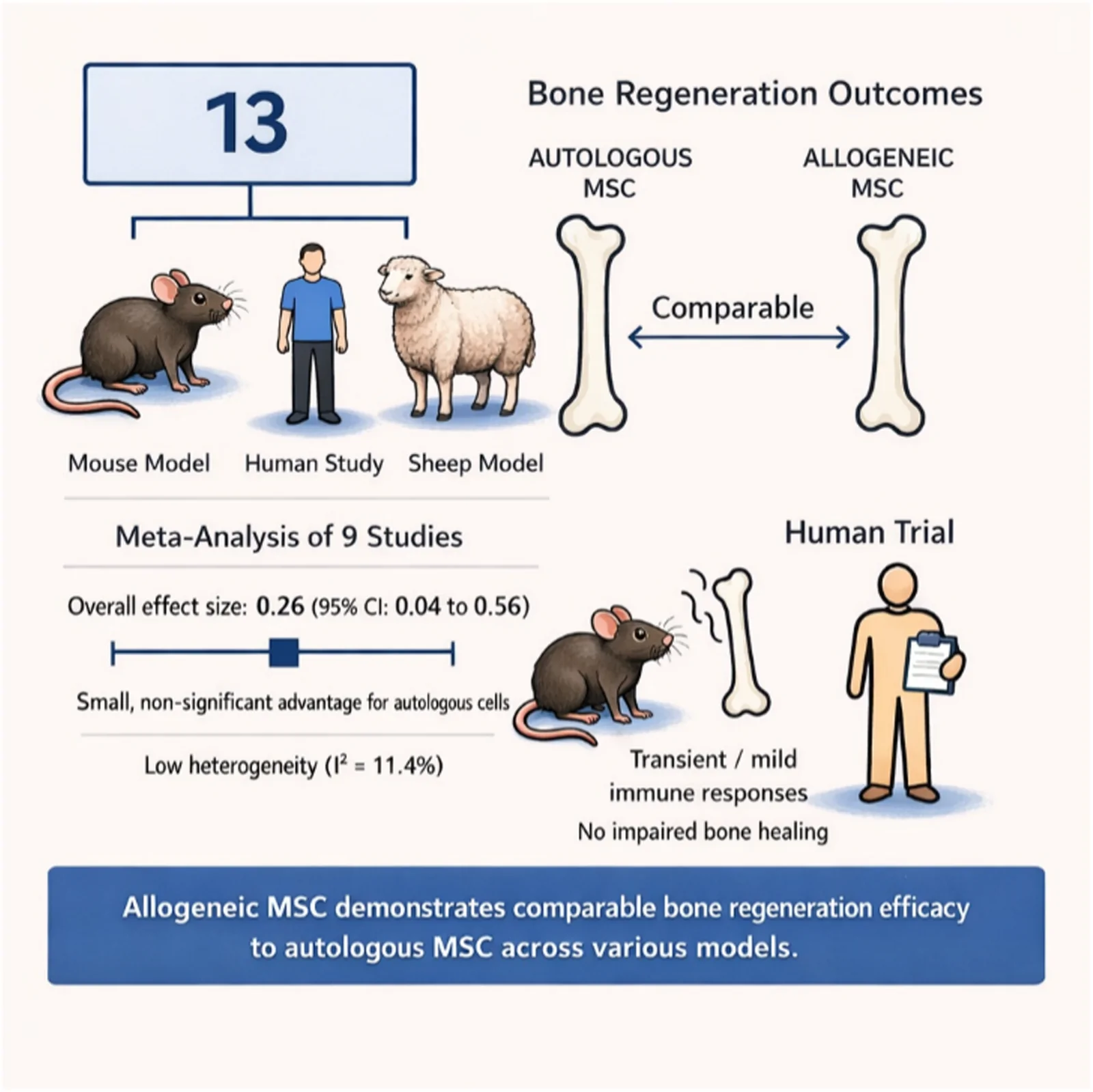
Illustrative overview of findings in the result section.

**Table 3.** Summary of study methods and outcome parameters evaluated.

Study	Species/defect site	Group (n)	Scaffold/ Carrier	Cell type	Cell dose	Cell modification/ treatment	Study period	Methods	Histology (staining)	Mechanical testing
Arinzeh et al., 2003^24^	Dog/Femur	Allo (4); Auto (6); NC (6)	Porous HA-TCP	BM-MSC	7.5 × 10^6^	Di-I labelling (CellTracker)	16 weeks	Radiography, histology, histomorphometry, allogeneic pair determination	H&E	Not reported
De Kok et al., 2003^25^	Dog/Alveolar	–	HA/TCP	BM-MSC (cryopreserved)	1,5 × 10^6^	Di-I labelling (CellTracker)	4 & 9 weeks	SEM, FC, histology	Goldner’s Trichrome; FITC	Not reported
Guo et al., 2009^16^	Mini-pig/Tibia	Allo (4); Auto (4); SCF (4); B (4)	β-TCP	BM-MSC	1 × 10^7^	Osteogenic induction, 3 weeks	16 weeks	FC, ELISA, RG	Not reported	Not reported
Kang et al., 2014^26^	Rabbit/Radius (bilat)	Allo (19); Auto (19)	Allogeneic cancellous bone granules	BM-MSC	2 × 10^6^	-	16 weeks	RG, µCT, histology	H&E; Masson’s trichrome	Not reported
Liu et al., 2013^27^	Dog/Cranial defect bilat	Allo (5); Auto (5); SCF (5)	Natural coral	AT-MSC	2 × 10^7^	Osteogenic induction, 3 weeks GFP labelling	24 weeks	MLRA, skin graft test, FC, ELISA, CT, µCT, histology	H&E; Azan-Mallory; GPF; Oil Red O; ALP; BSP; von Kossa	Not reported
Udehiya et al., 2013^28^	Rabbit/Radius	Allo (18); Auto (18); SCF (18)	HA	BM-MSC	3.45 × 10^6^	-	90 days	RG, angiography, histology	H&E	Weight bearing
Berner et al., 2013^29^	Sheep/Tibia	Allo (6); Auto (6); SCF (6); ABG (6)	mPCL-TCP/PRP	BM-MSC	35 × 10^6^	Osteogenic induction 2 weeks BrdU labelling	12 weeks	RG, CT, µCT, histology, IHC	H&E; von Kossa/McNeal’s; Goldner’s Trichrome	Torsion test, axial compression
Wu et al., 2016^30^	Dog/Mandible	Allo (3); Auto (3); Abone (3); SCF (3)	Natural coral; β-TCP	BM-MSC	20 × 10^6^	Osteogenic induction, 7 days	24 weeks	PCR-SSP, RG, CT, µCT, ELISA	H&E	3-point bending tests
Maiti et al., 2018^31^	Rabbit/Radius	Allo (6); Auto (6); Ovine (6); Canine (6); SCF (6); BLK (6)	HA-TCP	BM-MSC	6 × 10^6^	-	90 days	RG, SEM	H&E	Weight bearing
Rapp et al., 2018^32^	Humanized mice/Femur	Allo (8); Auto (4); SCF; Blank	Collagen gel	BM-MSC	2 × 10^5^	-	3, 20, 35 days	FC, µCT, Human Immunotherapy Panel, IHC, Histomorphometry	H&E; von Kossa	Not reported
Pan et al., 2021^17^	Rat/Tibia	Allo (10); Auto (10); NC (10)	None (injection in phosphate-buffered saline)	BM-MSC	5 × 10^5^	-	4 weeks	FC, µCT, RG, histology, double fluorescence, mechanical testing	H&E; von Kossa; Goldner trichrome	4-point bending tests
Chu et al., 2021^33^	Rat/Cranium	Allo (5); Auto (5); SCF (5); NC (5)	Gelatin-nanohydroxyapatite cryogel	BM-MSC	7.5 × 10^5^	Osteogenic induction, 7 days	12 weeks	FC, MLR, Ca & ALP stain, cell viability assay, µCT, histology, SEM	H&E; Masson’s trichrome	Not reported
Delclós et al., 2024	Human/Atrophic non-union long bones	Allo (6); Auto (6)	TCP	AT-MSC	6×10^6^- 56×10^6^ (depending on defect)	Osteogenic induction, 8 days	6 months	RG (CT when necessary)	Not reported	Clinical evaluation

Abbreviations: SFC – Scaffold; NC – Negative control; HA: hydroxyapatite; β-TCP: β-tricalcium phosphate; mPCL: medical grade polycaprolactone; PRP: Platelet-rich plasma; BLK – Blank; ABG – Autologous bone graft; BM-MSC – Bone marrow mesenchymal stem cells; MPC – Mesenchymal progenitor cells; BMD – Bone mineral density; BV – Bone volume; TV – Total tissue volume; H&E – Hematoxylin & Eosin; OCN – Osteocalcin; RUNX2 – Runt-related transcription factor 2; PECAM-1 – Platelet endothelial cell adhesion molecule 1; VEGFA – Vascular endothelial growth factor A; NA – Not applicable; ND – Not reported; NS – Not significant; SEM – Scanning electron microscopy; FC – Flow cytometry; PCR-SSP – PCR sequence specific primer; HU – Hounsfield units; BSP – Bone sialoprotein; BC – Bone coverage; BF – Bone fraction; FITC – Fluorescein isothiocyanate.

Cell delivery strategies varied across the included studies (Table 4). Only one study delivered MSC as a local injection of a cell suspension directly into/adjacent to the defect site without using a scaffold.^17^ In contrast, the majority of studies used scaffold-based delivery, where MSC were seeded onto or incorporated within a biomaterial scaffold prior to implanting into the defect. Biomaterial scaffolds utilized in pre-clinical studies, include hydroxyapatite-tricalcium phosphate (HA-TCP), Beta tricalcium phosphate (β-TCP), natural coral, gelatin-nanohydroxyapatite cryogel, and allogeneic bone granules, as well as more advanced composites such as medical-grade polycaprolactone-tricalcium phosphate (mPCL-TCP) and platelet-rich plasma (PRP). In the clinical trial TCP scaffolds were used for delivering cells.

**Table 4.** Summary of quantitative µCT, radiography and histomorphometry outcomes in autologous and allogeneic MSC-mediated bone regeneration.

Study	N (Auto)	µCT/Radiography (Autologous)	N (Allo)	µCT/Radiography (Allogeneic)	Histomorphometry (Auto)	Histomorphometry (Allo)	Effect on bone healing
Arinzeh et al., 2003^24^	4	1.56 ± 1.06 mm (Callus thickness)	4	0.75 ± 0.52 mm (Callus thickness)	42.3 ± 5.2 (% bone, 16w)	49.0 ± 12.3 (% bone, 16w)	Comparable healing
De Kok et al., 2003^25^	16	ND	16	ND	83% pores filled with bone	85% pores filled with bone	Comparable healing
Guo et al., 2009^16^	4	Subjective score	4	Subjective score	Not reported	Not reported	Comparable healing
Kang et al., 2014^26^	19	114.17 ± 24.38 mm^3^ (BV)	19	102.58 ± 12.81 mm^3^ (BV)	Not reported	Not reported	Comparable healing
Liu et al., 2013^27^	6	435 ± 115 mm^3^ (BV)** 76 ± 29 % bone coverage** 660 mg HA/cm^3^ (BD)**	6	395 ± 55 mm^3^ ** 73 ± 22 % ** 730 mg HA/cm^3^ (BD)**	Not reported	Not reported	Comparable healing
Udehiya et al., 2013^28^	18	11.00 ± 0.82* (subjective score)	18	11.00 ± 0.82* (subjective score)	Subjective score* 16.00 ± 4.1	Subjective score* 16.25 ± 1.50	Comparable healing
Berner et al., 2013^29^	—	Median values ∼ 1,000 mm^3^ **	—	Median values ∼ 1,300 mm^3^ **	Not reported	Not reported	Comparable healing
Wu et al., 2016^30^	3	1.65 ± 0.10 cm^3^ (BV) 0.30 ± 0.01 g/cm^3^ (BMD) 0.48 ± 0.01 g (BMC)	3	1.85 ± 0.15 cm^3^ (BV) 0.28 ± 0.02 g/cm^3^ (BMD) 0.50 ± 0.01 g (BMC)	Not reported	Not reported	Comparable healing
Maiti et al., 2018^31^	6	4.50 ± 0.45* (score)	6	4.02 ± 0.05* (score)	3.0 ± 0.23 (score)	2.7 ± 0.65 (score)	Comparable healing
Rapp et al., 2018^32^	4	1.65 ± 0.20 mm^3^** (BV) 36 ± 6.5 % bone area**	9	1.50 ± 0.40 mm^3^ **(BV) 16 ± 4 % bone area**	∼60% **	∼23% **	Allogeneic < autologous MSC
Pan et al., 2021^17^	10	+273.25 % (BV/TV); +334.76 % (BMD)	10	+279.76 % (BV/TV); +334.76 % (BMD)	1.12 ± 0.19 µm/day (MAR)	1.15 ± 0.17 µm/day (MAR)	Comparable healing
Chu et al., 2021^33^	5	93.5 ± 5.3 % (BV)	5	91.3 ± 1.1 % (BV)	Not reported	Not reported	Comparable healing
Delclós et al., 2024^34^	6	6/6 healed (subjective)	6	4/6 healed (subjective)	Not reported	Not reported	Comparable healing

Abbreviations: BV – bone volume; BC – bone coverage; TV – total volume; BMD – bone mineral density; BMC – bone mineral content; ND – not determined; HU – Hounsfield units; BQI – Bone quality index; MAR - Mineral apposition rate. * radiography data; ** values estimated from figures.

Across the included studies, MSC were primarily BM-MSC (n=11), with two studies using AT-MSC. However, among these included studies, the only clinical study used AT-MSC, not BM-MSC (Table 4). The administered cell dose varied substantially between the studies, depending on animal species, and defect models and sizes (Table 4). The lowest density in the preclinical studies was 2×10^5^ cells and the highest 35x10^6^ cells.^17,29^ For example, Rapp et al. seeded 2×10^5^ BM-MSC in a two-component type I collagen gel prior to implantation into the mouse bone defect. In contrast, in a large ovine segmental defect model, Berner et al. implanted 35×10^6^ bone marrow-derived mesenchymal progenitor cells (MPC) per construct. In the clinical study they used a cell dose ranging from 6×10^6^- 56×10^6^, depending on defect size.^34^

Among the included studies, 7 studies used non-differentiated (“naïve”) MSC,^17,24–26,28,31,32^ whereas 6 used stimulated osteogenic differentiation prior to implantation.^16,27,29,30,33,34^ Osteogenic induction was applied in both BM-MSC (4 studies) and AT-MSC approaches (2 studies). It is worth mentioning that the two studies that used AT-MSC applied osteogenic stimulation of the cells before implantation into animals. The stimulation time before implantation varied between 1-3 weeks. Three studies stimulated the cells for one week, one study used two weeks stimulation time, while two studies stimulated the cells for three weeks before *in vivo* implantation. Four studies used Di-I-labelled, GFP-labelled or BrdU-labeled cells to track the fate of the cells after *vivo* implantation.^24,25,27,29^

Follow-up duration varied substantially across the 13 studies, ranging from 4 weeks to 6 months. Most studies evaluated outcomes at medium-to-late time points (12-24 weeks/∼90 days-6 months), consistent with the time needed to capture meaningful bone maturation and remodeling.

### 3.3. Allogeneic MSC and immune response

Immune response outcomes were evaluated in the included studies using heterogeneous methods, including donor-recipient matching and *in vitro* immunogenicity testing (e.g., mixed lymphocyte reaction and/or circulating anti-donor antibody measurements), systemic immune profiling (pro-/anti-inflammatory cytokines and peripheral T-cell subsets such as CD4+/CD8+), and/or clinical safety monitoring (local/systemic inflammatory signs and adverse events). Two of the preclinical studies used only observation of the healing process and follow-up of the animals as an indicator for the immune response. In addition, the clinical study also used only clinical observation of the immune response. Out of the included studies, 2 preclinical studies relied only on observational/clinical assessment of the inflammatory response, such as wound appearance, swelling, infection or dehiscence, as their primary immune/safety signal.^28,31,34^ The rest of the preclinical studies included additional objective immune assessment, such as inflammatory cytokines concentrations and infiltration of inflammatory cells assessed by histology or immunohistochemistry.^16,17,24–27,29,30,32,33^ The specific cytokines reported were primarily T-cell and inflammation-associated markers, including interleukin 2, interleukin 4, interleukin 10, interferon gamma, transforming growth factor beta 1, and tumor necrosis factor alpha. For immune cell outcomes, several studies quantified peripheral T-cell subsets by flow cytometry, most commonly CD4-positive T cells and CD8-positive T cells. Local cellular responses were also assessed by histology/immunohistochemistry, including staining for CD8-positive T cells and F4/80-positive macrophages, and one study specifically evaluated CD4-positive lymphocyte infiltration at the regeneration site.^33^ Some studies additionally reported broader or indirect immune readouts, white blood cell counts, and humoral allo-antibody responses to allogeneic cells.^17,24–26,29,31,32^ Because these outcomes were evaluated using non-standardized and methodologically diverse approaches, ranging from clinical observation to *in vitro* immunoassays such as the mixed lymphocyte reaction, they are not directly comparable and are synthesized narratively below rather than pooled quantitatively.

Overall, no meaningful difference in immune response between autologous and allogeneic cell groups (no systemic response, no signs of rejection, or comparable immunologic readouts) were reported,^16,17,24–29,31,34^ whereas three detected a mild/transient immune activation in the allogeneic groups (typically early cytokine/T-cell changes or mild cellular responses) in humanized mice, minipigs and dogs.^16,30,32^ Importantly, none of the studies reported signs of severe or persistent immune reaction leading to rejection or graft failure.

In the clinical study particularly, although relying only on clinical observation, comparable responses without clinical signs of local or systemic reactions indicating rejection were reported for both autologous and allogeneic cell groups. Though two treatment failures (non-unions) were reported, none of these were reported as being caused by an immune reaction.

### 3.4. Allogeneic MSC effect on bone formation

For evaluation of bone formation, various methods were implemented. Nine of the studies reported radiographic analysis of bone formation (Table 5). In the included studies, imaging was a commonly used outcome measure, but there was substantial variation in the choice of modality and in how quantitative the analyses were. Conventional radiography (plain X-ray) was used in the majority of studies (9 of 13),^16,17,24,26,28–31,34^ often as semi-quantitative scoring or as a clinical assessment of union. µCT was used in about half of the studies (7 of 13)^17,26,27,29,30,32,33^ and provided more detailed, quantitative measures of bone regeneration such as bone volume (BV), BV/TV, and bone mineral density (BMD). Clinical CT was reported in fewer studies (4 of 13),^27,29,30,34^ typically as an adjunct to radiography or on an as-needed basis, particularly in large-animal models and in the clinical study. Some studies employed scoring systems, either previously reported/established indices (e.g., BQI) or study-specific subjective grading, for reporting their findings. Three studies assessed vascularization in parallel with osteogenesis, based on angiography, VEGFA and the endothelial marker CD31 and Cyr-61, for quantification of angiogenic activity within the regenerated bone.^17,28,32^

**Table 5.** Immune response assessment.

Study (year)	Immune assessment (methods)	Main immune response/safety finding
Arinzeh et al., 2003^24^	Flow cytometry analysis of humoral allo-antibody response to allogeneic cells, and histology	No systemic or local immune response to allogeneic MSC.
De Kok et al., 2003^25^	Flow cytometry analysis of humoral allo-antibody response to allogeneic cells, and histology	Absence of systemic or local immune response to allogeneic MSC.
Guo et al., 2009^16^	Flow cytometry for T-cell subsets (CD4+, CD8+) in blood and ELISA for IL-2, IL-10 levels in serum	Allogeneic MSC showed little/no immunogenicity without significant differences from autologous MSC.
Kang et al., 2014^26^	Histological evaluation	No evidence of adverse immune reactions such as inflammatory cell/macrophage infiltration in autologous and allogeneic groups.
Liu et al., 2013^27^	Flow cytometry for T-cell subsets (CD4+, CD8+) in blood, cytokine profile (IL-2, IL-4, IL-10, IFN-g and TGF-b1) in serum, histology and immunohistochemistry staining against the GFP-labelled cells	Similar systemic and local immune responses in autologous and allogeneic groups.
Udehiya et al., 2013^28^	Only observation of healing at the defect site and follow up of the animal	Uneventful healing, without signs of rejection.
Berner et al., 2013^29^	Blood samples for basic analysis of immune reaction (e.g., white blood cell count), histology and immunohistochemistry staining against the BrdU-labelled cells	Comparable results from blood samples testing, with no clinical signs of undesirable immune reaction or graft rejection.
Wu et al., 2016^30^	Flow cytometry for T-cell subsets (CD4+, CD8+) in blood, cytokine profile (TNF-α, IFN-γ, IL-2, IL-10) in serum	Transient early immune response in allogeneic group; no clinical signs of rejection, suggesting allo-BM-MSC not fully immunoprivileged but clinically well tolerated.
Maiti et al., 2018^31^	Only observation of healing at the defect site and follow up of the animal	No signs of undesirable inflammatory reaction or complications reported.
Rapp et al., 2018^32^	Multiplex analysis of inflammatory cytokines in serum, Immunohistochemistry staining of CD8+ T cells and F4/80+ macrophages	Mild cellular immune response to allogeneic MSC; did not prevent bone regeneration.
Pan et al., 2021^17^	Histological evaluation	No inflammatory callus or inflammatory cell infiltration was observed, indicating no immunological rejection
Chu et al., 2021^33^	Immunohistochemistry staining of CD4+ T cells	No CD4+ lymphocyte infiltration along the regenerated bone in either allogeneic or autologous BM-MSC group, indicating the absence of CD4+-mediated immune response.
Delclós et al., 2024^34^	Clinical evaluation during follow-up	No serious adverse events; allogeneic MSC judged clinically safe.

Six studies included histomorphometric analysis, 5 of them used histomorphometric analysis together with Radiographic evaluation, while one study used only histomorphometry for evaluation of bone formation. Histomorphometric evaluation was heterogeneous across the included studies, ranging from purely descriptive histology to quantitative static/dynamic histomorphometry. Histomorphometry data were reported as percentage in some studies, whereas in other studies scoring systems were followed (Table 4).

Regarding bone regeneration, 11 preclinical studies concluded that allogeneic MSC supported bone regeneration comparable to autologous MSC at the final evaluation time point. Only one preclinical study reported inferior regenerative outcomes for allogeneic MSC, mainly reflected by reduced bone volume and histomorphometric parameters.^32^ Overall, most studies demonstrated successful defect healing and comparable bone formation between autologous and allogeneic MSC, despite the presence of a detectable immune response in a subset of studies.

The preclinical studies that evaluated bone formation using radiographical analyses, including X-ray, CT and/or µCT, reported comparable increases in bone formation parameters like bone volume, bone/tissue volume and bone mineral density. Also, in the studies adopting scoring systems for evaluation of bone formation and healing similar findings were reported.

For evaluation of bone quality, three studies implemented mechanical testing that included torsion test, axial compression in addition to 3- and 4 point bending tests^17,29,30^ (Table 4). Three preclinical studies conducted mechanical testing, such as compression, 3- and 4-points bending tests, of the regenerated bone and provided further insights into its structural integrity.^17,29,30^ The biomechanical testing revealed maintained mechanical stability with no significant difference between the autologous and the allogeneic group, although quantitative data were inconsistently reported across studies.

Using various methods, including angiography, immunohistochemical staining for VEGFA and the endothelial marker CD31 and Cyr-61, 3 preclinical studies evaluated vascularization of the newly formed bone and found no differences between the allogeneic and autologous groups in vascularity and vessel formation within the regenerated bone.^17,28,32^

Four studies used different types of cell labelling, such as Di-I-labelled, GFP-labelled or BrdU-labeled cells,^24,25,27,29^ to track the fate of the implanted cells during the bone formation process. Three of the studies, using Di-I and GFP labeling found that both autologous and allogeneic MSC were detected in the newly regenerated bone, with marked cells at different time points up to 12 weeks, whereas one study did not detect any BrdU-positive cells, i.e., no evidence of donor-cell survival at 12 weeks.^29^

The single human study that was included,^34^ assessed bone healing primarily by radiography and with computed tomography when necessary, and follow-up focused on clinical and safety outcomes. Overall, bone healing was achieved in 10 of 12 cases (83%). Radiographic union was reported in 6 of 6 patients in the autologous AT-MSC group and 4 of 6 in the allogeneic AT-MSC group. The authors also noted that the overall healing rate was comparable to a historical cohort treated by the same team using a fixed dose of autologous bone marrow derived mesenchymal stromal cells.

### 3.5. Meta-analysis

Nine *in vivo* studies provided sufficient quantitative data to be included in the meta-analysis. The pooled effect size (Hedges’ g) slightly favored the autologous group, with an overall effect size of 0.26 (95% CI: [0.04, 0.56]), indicating a positive, but small effect in their favor (Figure 5). However, the prediction interval (95% PI: [−0.27, 0.79]) suggests considerable uncertainty regarding the true effect on future studies (Figure 4).

**Figure 5.**
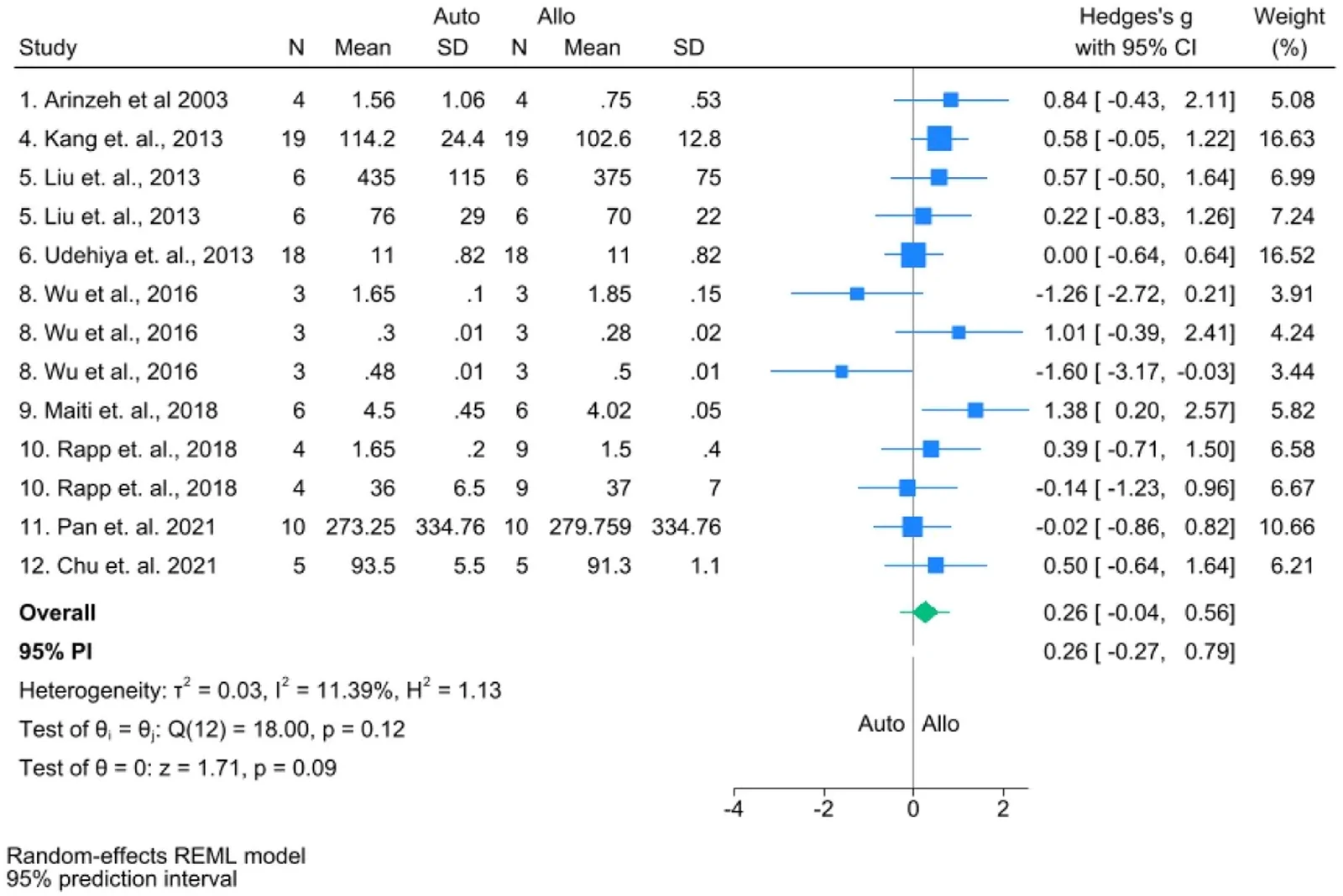
Forest plot comparing autologous (Auto) and allogeneic (Allo) MSC for bone regeneration. Each study is represented with its standardized mean difference (Hedges’ g) and 95% confidence interval. Positive values favor autologous MSC. The overall effect size was 0.26 (95% CI: 0.04 to 0.56), indicating a small, non-significant advantage for autologous cells. Heterogeneity was low (I^2^ = 11.4%), and the 95% prediction interval ranged from –0.27 to 0.79.

Across the 13 studies included in this meta-analysis, heterogeneity was low (τ^2^ = 0.03, I^2^ = 11.39%) as shown in Figure 5, indicating relatively consistent results across studies. The Q-test for heterogeneity was not significant (Q (12) = 18.00, p = 0.12), and the test for the overall effect did not reach conventional statistical significance (z = 1.71, p = 0.09), suggesting that there might be a trend toward benefit with autologous MSC.

The funnel plot (Figure 6) showed slight asymmetry, possibly indicating publication bias or small-study effects. However, the overall distribution was relatively symmetric around the estimated average effect size.

**Figure 6.**
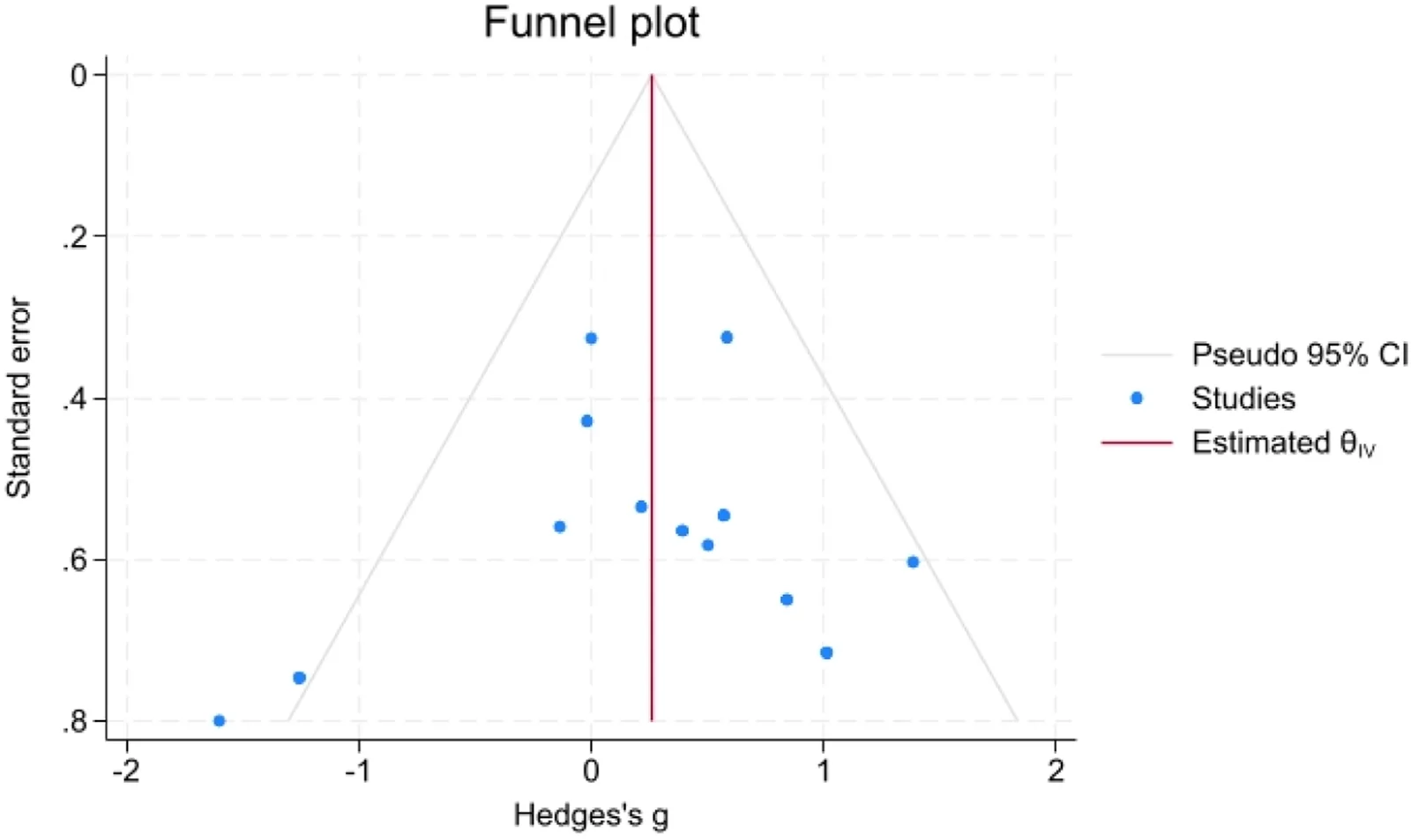
Funnel plot assessing publication bias in studies comparing autologous and allogeneic MSC. Each dot represents a study’s effect size (Hedges’ g) plotted against its standard error. The symmetrical distribution of points within the pseudo 95% confidence limits suggest minimal risk of publication bias.

Sensitivity analyses (leave-one-out analyses) showed that the overall results were robust, as no single study had a disproportionate influence on the pooled estimate (Figure 7). Removal of any individual study did not substantially change the overall effect size, which ranged from 0.20 to 0.32 across all iterations.

**Figure 7.**
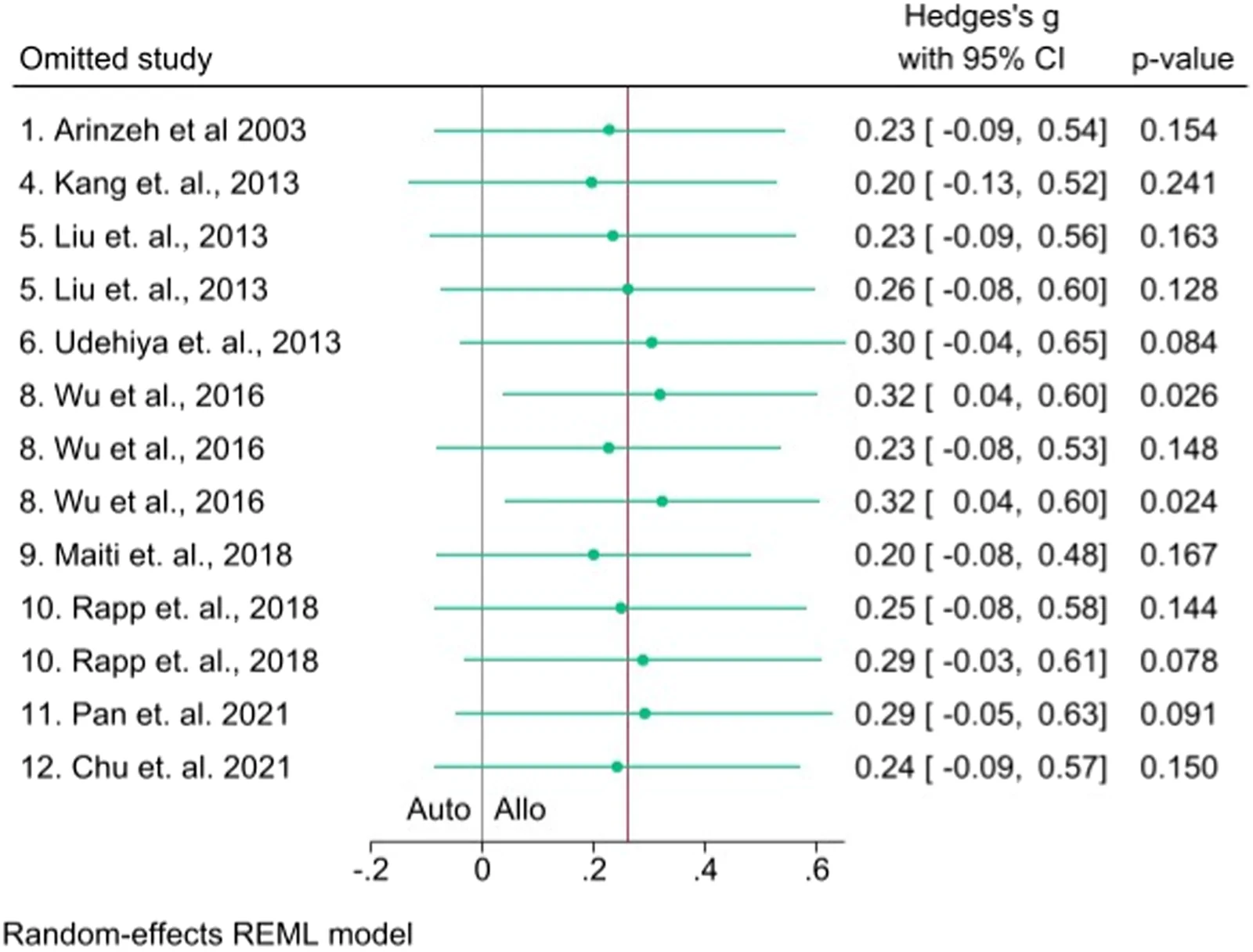
Sensitivity (leave-one-out) analysis showing the pooled effect size after omitting one study at a time. The overall effect remains stable (range: 0.20 to 0.32), indicating that no single study disproportionately influenced the meta-analysis result.

In summary, the meta-analysis revealed a small effect favoring autologous MSC for bone regeneration, but the differences were not statistically significant. The findings support the conclusion that allogeneic MSC perform comparably to autologous MSC in most preclinical models, reinforcing their potential as a viable alternative in clinical applications. Of the nine studies included in the quantitative meta-analysis, 3 used osteogenically primed MSC^27,30,33^ prior to implantation and 6 used non-differentiated (naïve) MSC.^17,24,26,28,31,32^ Because osteogenic priming alters the differentiation state and immunomodulatory profile of MSC, this represents a potential confounding variable when comparing autologous and allogeneic cell sources. An explicit statement that the available sample size was insufficient to support a formal quantitative subgroup analysis, with priming status instead discussed narratively in Section 4.4.1.

## 4. Discussion

The use of allogeneic MSC for the reconstruction of bone defects has shown promising potential, especially when compared to traditional methods such as autologous grafting.^35^ The findings from several studies, including those discussed in this systematic review, provide insight into the advantages and challenges of using allogeneic MSC in bone regeneration.

One of the most notable advantages of allogeneic MSC is their immediate availability, which distinguishes them from autologous MSC that require invasive harvesting procedures and time-consuming *ex vivo* expansion. The ready availability of allogeneic MSC enables rapid clinical deployment, which is particularly valuable in managing extensive bone defects where timely intervention is important to restore both function and aesthetics. This immediacy not only facilitates emergency surgical planning but also enhances patient accessibility to regenerative therapies without the delays associated with autologous cell preparation. Furthermore, the logistical convenience of allogeneic MSC may contribute to broader clinical adoption and scalability, especially in settings where autologous harvesting is contraindicated or impractical. However, while their availability is advantageous, it must be weighed against concerns regarding immunogenicity and long-term safety, which remain active areas of investigation.

### 4.1. Influence of biomaterial scaffolds on regenerative outcomes

The following section provides contextual background on biomaterial scaffold technologies relevant to the field of bone tissue engineering. With the exception of the specific scaffold types used within the included studies (summarized in Table 3), the broader scaffold literature discussed below was not directly evaluated within this systematic review and is presented to inform interpretation and future research directions, rather than as a finding derived from the reviewed studies.

Scaffold-based delivery was the predominant strategy across the included studies, most often pairing MSC with an osteoconductive carrier to improve local cell retention, provide a 3D niche, and concentrate regenerative activity within the defect. In contrast, injection-based delivery represents a simpler approach, but may be associated with less predictable cell retention, dispersion, and localization at the target site.

In bone-tissue-engineering studies, biomaterial scaffolds are essential companions to MSC in bone regeneration, as they maintain defect volume and provide a 3D environment that supports cell attachment, survival and differentiation.^36^ Their physicochemical properties such as porosity, surface topography, mechanical strength and degradation rate influence osteogenic differentiation and subsequent bone formation.^36^ In the included studies in this systematic review, a range of scaffold materials was used spanning calcium phosphate ceramics (HA-TCP, β-TCP), naturally derived matrices (coral and allogeneic bone granules), and polymer/biologic adjuncts, including gelatin-nanohydroxyapatite cryogels, medical-grade mPCL-TCP composites, and platelet-rich plasma (PRP). Calcium-phosphate ceramics such as HA-TCP and β-TCP resemble bone mineral, support MSC attachment/osteogenic differentiation, and have been widely used to promote ectopic or orthotopic bone formation; however, their remodeling kinetics and brittleness can limit performance, particularly when resorption and new bone formation are not well matched or when higher mechanical demands exist.^37–41^ Natural coral offers a highly interconnected porous architecture that can support MSC proliferation and enhance healing in segmental defect models, but as a naturally sourced material may raise concerns about batch variability and the need for processing/phase conversion.^42^ Gelatin-nanohydroxyapatite cryogels add cell-friendly, ECM-like cues and tunable mechanics/porosity, and design features (chemical/physical cues) can further enhance MSC osteogenesis, although hydrogel-based constructs may require reinforcement for load-bearing applications.^43^ Allogeneic bone granules provide native bone matrix cues and can serve as an MSC carrier, but biological performance can vary with donor factors and processing/storage methods.^26,44^ More advanced composites like mPCL-TCP improve shape fidelity and mechanical stability while leveraging TCP osteoconductivity, yet the polymer component may degrade slowly and potentially prolong the presence of foreign material.^45,46^ Finally, PRP can provide a growth-factor-rich milieu and may synergize with MSC, but variability in PRP preparation and release kinetics likely contributes to inconsistent outcomes; combining PRP/MSC within composite scaffolds has shown promising preclinical/clinical-direction signals in implant-associated regeneration contexts.^47–49^ All these findings underline the importance of scaffold choice for successful osteogenesis.

Generally, for clinical bone regenerative applications, the combination of MSC with calcium phosphate-based scaffolds, for example biphasic calcium phosphate (BCP) granules, has repeatedly shown favorable bone regeneration in preclinical^25,50,51^ as well as clinical settings.^6,52,53^

3D-printed and defect-specific scaffolds from calcium phosphate-based materials have emerged as a promising refinement, allowing control over geometry and porosity and the incorporation of other bioactive agents to better match mechanical and biological requirements of complex bone defects, for example jawbone defects.^54,55^ Polymer ceramic composites (e.g. PCL or PLA combined with HAp/β-TCP) and customized printed scaffolds have shown encouraging results in preclinical models and early clinical applications such as alveolar ridge augmentation, although issues such as mechanical weakness in highly porous constructs, mismatch between degradation and bone formation, and limited vascularization in large defects remain important challenges that future studies must address.^56,57^ In 3D scaffold systems relevant to bone regeneration, the scaffold/cell interplay is a key determinant of performance, and the origin of the MSC (autologous vs allogeneic) may therefore influence the final regenerative outcome.^58^

### 4.2. Safety of allogeneic MSC compared to autologous MSC

Safety is a central consideration for translating allogeneic MSC-based bone regeneration into clinical use. The safety of MSC-based bone regeneration is usually assessed in two complementary ways: general/systemic safety and local/on-target and off-target safety. GLP-style studies of allogeneic MSC products in rodents and large animals typically monitor survival, behavior, weight gain, food intake, clinical signs, and standard hematology and serum chemistry panels, followed by gross necropsy and histopathology of major organs.^59^ Biodistribution studies using labelled MSC (e.g. fluorescent, radionuclide or qPCR detection of human DNA) are performed to determine how long cells persist and whether they accumulate in non-target organs such as lung, liver or spleen.^60^ Tumorigenicity is typically addressed by long-term follow-up in immunodeficient mice receiving supratherapeutic doses of the MSC product (sometimes including genetically modified or cytokine-primed variants), with systematic histological screening for mesenchymal tumors or ectopic tissue formation.^61^ In bone regeneration models, additional local safety endpoints include the presence of chronic inflammation, foreign-body reactions or osteolysis at the defect site, as well as ectopic ossification in adjacent soft tissues or distant organs as detected by µCT and histology.

A further safety aspect is the biological age and stability of the MSC product. Prolonged culture expansion drives MSC senescence, with changes in morphology, reduced differentiation capacity and a senescence-associated secretory phenotype that can promote chronic inflammation and alter immunomodulatory behaviour.^62^ Senescent MSC may activate innate and adaptive immune responses more strongly, which is undesirable in allogeneic settings. This underlines the importance, even in animal studies, of strict *in vitro* quality control: limited passage number, karyotyping, assessment of senescence markers, and functional immunomodulation assays before implantation.^62^

Despite the advances seen with allogeneic MSC, autologous MSC are still regarded as the gold standard for bone regeneration due to their great osteogenic potential, favorable safety profile, and lack of immunogenicity concerns.^63^ Autologous cells offer a biologically compatible source that minimizes the risk of immune rejection and disease transmission while maintaining robust differentiation and signaling within the host microenvironment.^64^ Large clinical meta-analyses of autologous MSC therapy across diverse indications show no increase in serious adverse events such as death, infection, organ complications or malignancy compared with controls, although transient fever, local injection-site reactions, constipation and fatigue occur somewhat more frequently.^65^ Similar findings were reported in an earlier systematic review, which also concluded that intravascular MSC therapy appears safe when standard dose ranges and routes are used.^66^ However, the use of autologous MSC is not always feasible. Factors such as patient age, comorbidities, and limited availability of healthy donor tissue can significantly affect the yield, viability, and quality of the harvested cells.^67^ Moreover, the time required for cell isolation, expansion, and preparation presents logistical challenges, particularly in acute or large bone defects where immediate treatment is needed.^68^

In this context, allogeneic MSC represents a promising alternative. They can be sourced from young, healthy donors in large, standardized batches, allowing ready availability and consistent therapeutic quality.^69^ Nevertheless, despite their clinical practicality, the immunological and functional equivalence of allogeneic to autologous MSC remains area of active debate. Studies indicate that the immunomodulatory properties of MSC reduce the risk of rejection, and in several cases, allogeneic MSC can be used without the need for immunosuppressive treatment.^64,70^ Nevertheless, it is essential to consider individual patient factors, host immune competence, and optimal administration protocols to maximize therapeutic efficacy.^71^ To our knowledge to date, systematic reviews with meta-analysis that focus mainly on the safety of allogeneic MSC for bone regeneration are lacking, but the studies that have been conducted nevertheless indicate that allogeneic MSC can be safe when used clinically for therapeutic applications.^34,72^

Although MSC are often described as “immune privileged,” accumulating evidence indicates that they can elicit immune responses under certain conditions, which may affect their persistence, engraftment, and regenerative efficacy.^73^ Depending on the inflammatory microenvironment, allogeneic MSC may upregulate MHC class II or provoke T cell-mediated responses.^74^ To overcome these limitations, several strategies have been explored, including biomaterial scaffolds that provide immunoprotective niches or spatial isolation of the cells,^75^ genetic or preconditioning approaches (e.g., hypoxia, cytokine priming, gene modification) to enhance MSC survival and paracrine signaling,^76^ and co-delivery with immunomodulatory agents (e.g., regulatory T cells).^77,78^

Because immune-related complications remain a principal barrier to the clinical translation of allogeneic cell therapies, several complementary strategies for mitigating MSC immunogenicity merit further discussion. Donor selection and cell-banking approaches, including partial HLA matching and screening for low baseline MHC expression, may reduce the risk of alloimmune sensitization,^79^ although this adds logistical complexity to an “off-the-shelf” model. At the cellular level, *ex vivo* licensing of MSC with inflammatory cytokines such as IFN-γ or TNF-α, or exposure to hypoxic preconditioning, has been shown to modulate MHC class II expression and enhance immunosuppressive pathways (e.g., indoleamine 2,3-dioxygenase and prostaglandin E2 production), potentially improving graft tolerance before implantation.^80,81^ Genetic engineering approaches, such as CRISPR-based knockout or knockdown of MHC class I and/or class II expression (e.g., via disruption of β2-microglobulin or CIITA), have been explored to generate “hypoimmunogenic” MSC with reduced alloreactivity and improved *in vivo* survival, although the long-term safety and regulatory implications of such modifications remain to be established.^79,82^ Biomaterial-based strategies represent a further avenue relevant to bone regeneration specifically: encapsulation of MSC within immunoprotective hydrogels or semi-permeable scaffolds may physically shield cells from direct immune cell contact while still permitting diffusion of therapeutic paracrine factors, and scaffolds capable of local, sustained release of immunomodulatory or anti-inflammatory agents could dampen local immune activation without the systemic side effects of pharmacological immunosuppression.^83^ Finally, co-delivery with regulatory T cells or other immunomodulatory cell populations has been proposed as a complementary cellular strategy to promote tolerance at the implantation site.^77,78^ None of these strategies were directly evaluated within the studies included in this review, and their comparative efficacy specifically in bone regeneration remains largely untested. Incorporating standardized immunogenicity endpoints, such as donor-specific antibody titers, mixed lymphocyte reaction assays, and longitudinal T-cell profiling, into future preclinical and clinical trials of allogeneic MSC for bone regeneration would help clarify which, if any, of these mitigation strategies meaningfully improve safety and efficacy outcomes, providing a clearer roadmap toward successful clinical translation.^75^

Based on the data from the preclinical studies included in this review, generally allogeneic MSC did not induce meaningful difference in immune response or show signs of persistent immune reaction leading to rejection, when compared to autologous MSC. Allogeneic MSC could integrate into host tissue, even without immunosuppressive treatment. The absence of undesirable local or systemic immune response for both allogeneic and autologous MSC was reported in various models and in different species. In small animals (rat and rabbit), there was no evidence of adverse immune reactions such as inflammatory cell/macrophage infiltration in autologous and allogeneic groups, indicating no immunological rejection.^17,26,28,31–33^ Likewise, in large animals (dog, minipig and sheep), similar local and systemic immune responses in autologous and allogeneic groups with no clinical signs of undesirable immune reaction or graft rejection were observed.^16,24,25,27,29,30^ However, slightly different findings were described in other studies showing that allogeneic MSC triggered a transient immune response and initial immune activation that led to slightly delayed osteogenesis compared to autologous MSC; but this rarely affected the long-term outcome of bone regeneration.^24,27,30^ For example, in dogs, Wu et al. observed an increase in pro-inflammatory cytokines (TNF-α, IFN-γ, IL-2) and a decrease in anti-inflammatory markers (IL-10) in the first two weeks after implantation. This coincided with an increased proportion of CD4+ and CD8+ T-cells in peripheral blood; however, these levels normalized over time, and bone regeneration at 24 weeks was comparable to that of autologous MSC.^32^ In another study, Rapp et al. also demonstrated that when the immune environment is modeled more realistically (humanized mice), allogeneic MSC elicited a mild early local immune response (increased CD8^+^ T cells), and at later time points showed inferior regenerative efficacy.^32^

Like most of the included preclinical studies reporting uneventful healing, without signs of systemic or local immune response to allogeneic MSC, Delclós-Orozco et al., in their clinical study, showed comparable responses without clinical signs of local or systemic signs indicating rejection for the autologous as well as the allogeneic groups, although relying only on clinical observation.^34^ They treated 12 patients with various types of bone defects with autologous (6) or allogeneic AT-MSC (6) with a cell dose ranging from 6×10^6^ to 56×10^6^ AT-MSC, depending on defect size, and patients were followed for 12 months for safety outcomes. They reported two treatment failures (non-union) cases using allogeneic MSC in this clinical study, but they concluded that these failures were not caused by an immune reaction. These data provide strong evidence for the safety of allogeneic MSC for treatment of bone defects/fractures.

Taken together, these data support the overall safety of allogeneic MSC as a therapeutic class, but they do not replace the need for additional rigorous non-clinical as well as clinical safety testing.

### 4.3. Efficacy of allogeneic MSC in bone regeneration compared with autologous MSC

All included studies were direct head-to-head comparisons of autologous versus allogeneic MSC, consistent with our inclusion criteria. Across donor sites, both the individual studies and our meta-analysis show that bone regeneration is largely comparable between autologous and allogeneic MSC, with a small, non-significant advantage for autologous cells. Across preclinical literature, this overall comparability is remarkably consistent despite the use of heterogeneous animal models and defect settings. Studies directly comparing small animals (rat and rabbit)^17,31,33^ and large animals (dog, sheep and minipig)^27,29,30^ generally report similar radiographic/µCT-derived bone formation, mineralization and/or histological healing in autologous and allogeneic MSC groups at the final follow-up, even when constructs differ in anatomical site (craniofacial vs long bone), defect type (critical-sized defects, segmental defects, distraction osteogenesis), and carrier materials (e.g., calcium phosphate ceramics, coral, allograft granules, polymer-ceramic composites).

The studies that conducted µCT and/or radiographic data analysis reported that BV and/or BV/TV and bone mineral density significantly increased in most studies. The allogeneic groups exhibited comparable bone formation, though in some cases, slightly lower values than their autologous counterparts. For example, using a radius defect model in rabbits, Kang et al. reported BV values of 114.2 ± 24.4 mm^3^ in autologous grafts, compared to 102.6 ± 12.8 mm^3^ in allogeneic grafts.^26^ Interestingly, slightly higher values of BV and/or BV/TV in the allogeneic group than in the autologous group have also been reported in some studies. For instance, Wu et al. studied bone regeneration in the mandible of dogs and reported 1.65 ± 0.10 cm^3^ BV for the autologous group, while it was slightly higher for the allogeneic group with 1.85 ± 0.15 cm^3^ BV.^30^ Similarly, Pan et al. observed a 273,25% increase in BV/TV in the autologous group compared to the control (treated with only phosphate buffered saline (PBS)), whereas the allogeneic group showed an increase of 279,76% in a distraction osteogenesis model in rats tibia.^17^ In their study, Pan et al. further analyzed mineral apposition rate (MAR) reporting values of 1.15 ± 0.17 µm/day for allogeneic grafts, and 1.12 ± 0.19 µm/day and 0.28 ± 0.02 g/cm^3^ for autologous grafts, suggesting similar bone growth kinetics between the two treatment groups. They also found comparable bone mineral density (BMD) in the two groups.^17^ A similar observation regarding BMD was also noted in another study after treating the defects with allogeneic or autologous cells.^27,30^ For comparing bone regeneration after treatment with autologous or allogeneic MSC constructs, a group of studies relied on subjective radiographic scoring systems for assessments of bone regeneration and reported comparable scores for bone regeneration in long bone defect models in rabbits and minipigs.^16,28,34^ However, the scoring approach was inconsistently described across papers and described as either previously reported/established indices (e.g., Bone Quality Index (BQI)) or study-specific subjective grading, limiting cross-study comparability. For example, Kang et al. used a radiographic BQI based on defect filling and bone density in a rabbit radial defect model and found increasing scores over time in both autologous and allogeneic MSC groups, although the allogeneic group showed slightly lower BQI at later time points.^26^ In addition to this, Guo et al. shows graded radiographic healing using a 0-4 subjective score for bone formation, synostosis, and bone modelling rather than a standardized index, and found similar bone-healing scores in autologous and allogeneic MSC-treated minipigs.^16^

A set of studies used histological/histomorphometric analysis for evaluation of bone regeneration, either alone^25^ or complementing radiographic analysis,^17,24,26–33^ also showing similar bone regeneration by autologous and allogeneic MSC. However, reporting was also inconsistent among the studies, and largely descriptive rather than with standardized numeric histomorphometric readouts. For example, Udehiya et al. applied a semi-quantitative histological score for osteogenesis, union, marrow, cancellous bone, and cortical bone, and reported no significant difference between autologous and allogeneic MSC-treated rabbit radius defects.^28^ Another study by De Kok et al. used histomorphometrical analysis based on the percentage of scaffold pores filled with bone or osteoid on Goldner’s trichrome sections and found similar bone formation in autologous and allogeneic MSC groups (83% vs. 85% of pores filled, respectively) in a canine alveolar defect model.^25^

With regard to bringing allogeneic MSC directly into clinical practice, the study by De Kok et al.^25^ addresses an important aspect that could make this feasible. De Kok et al. demonstrate the direct use of allogeneic MSC immediately on thawing after cryopreservation, which represents an optimal use^25^. In their study, they loaded HA/TCP matrices with cryopreserved allogeneic or autologous MSC one day prior to implantation in an alveolar bone defect model in dog mandibles. They also labelled these cells with Di-I (CellTracker) to track the fate of these cells after *in vivo* implantation. They evaluated bone formation histologically based on the percentage of filled pores with bone or osteoid revealing that both autologous and allogeneic MSC-loaded constructs had a high percentage of pores filled with bone for autologous MSC group (83%) and allogeneic MSC group (85%) compared to the cell-free group, with 60% of pores filled with bone. They also detected fluorescence signals of the labelled autologous as well as allogeneic MSC adjacent to osteoid and within the lacunae of the newly formed bone, confirming the survival of implanted MSC, and indicating their contribution to the bone formation process. The ability to avoid the need for expansion in culture would facilitate their clinical use and reduce the associated time and expense.

In total, four studies in this review used different types of cell-labelling to track the fate of the cells after *in vivo* implantation, including Di-I -(CellTracker)-, BrdU- and GFP-labelled cells.^24,25,27,29^ Three of the studies in dogs were able to demonstrate the implanted MSC in new bone formation at the end of their follow-up period.^24,25,27^ This provides compelling evidence that the transplanted MSC can survive and potentially participate directly in the process of bone formation and differentiation. These observations are relevant to the ongoing discussion of whether MSC promote regeneration directly (by engraftment and differentiation into bone-forming cells and producing bone matrix) or indirectly (through stimulating the host’s own cells by trophic/paracrine and immunomodulatory effects). The persistence of labelled cells within regenerated tissue supports a potential direct cellular contribution, but it does not exclude an important (or even predominant) paracrine mechanism, which has been widely proposed as a central mode of MSC action in tissue repair.^84,85^ With this issue in mind, Rapp et al. implanted allogeneic and autologous MSC in femoral defects in humanized mice, and after conducting species-specific collagen staining, they found that the newly deposited bone matrix was of murine origin, suggesting that transplanted hMSC contributed predominantly via indirect mechanisms rather than directly synthesizing bone matrix.^32^ Importantly, not all tracking studies detected donor cells at later stages: in the included study in sheep by Berner et al., BrdU-labelled cells were not detectable at 12 weeks, and the authors suggested that label dilution during cell expansion/proliferation could contribute to false-negative detection at endpoint.^29^

The relative contribution of direct versus indirect mechanisms likely depends on multiple factors, including the source and preconditioning of the MSC, the local microenvironment, the type of scaffold used, and the immune status of the host. These mixed findings underscore that the extent and durability of allogeneic MSC persistence and their direct versus indirect contribution to bone regeneration remain uncertain and should be explored further across models, timepoints, and detection methods.

A few studies observed weight bearing and further evaluated the quality of healing and the formed bone by conducting mechanical testing of the bones.^17,29,30^ One study in sheep showed no significant differences in torsional stiffness between autologous and allogeneic cell groups, while torsional moment values were marginally higher in the autologous group, though not statistically significant.^29^ Another study in rabbits conducted 4-point bending test by load-to-failure test recording maximum load, energy to failure and stiffness.^17^ These tests showed that bones with allogeneic MSC performed comparably to their autologous counterparts, with no statistically significant differences observed. From conducting 3-point bending tests on the mandibles from the dogs after treatment with allogeneic or autologous MSC, Wu et al. also reported generally similar values for ultimate load, bending strength and Young’s modulus.^30^

Since vascularization plays an important role during the bone formation process, by providing oxygenation, nutrition and waste removal, three of the included studies additionally examined vascular formation within the formed bone using angiography^28^ and immunohistochemical staining for the angiogenic factor VEGFA and the endothelial marker CD31.^17,32^ After implanting allogeneic or autologous MSC together with HA in radius defect model in rabbits, Udehiya et al. evaluated vascularization using angiography.^28^ They reported comparable bone healing with intense vascularity at the defect sites treated with either allogeneic or autologous MSC, while in a control cell-free group, the vascularization was minimal. In their distraction osteogenesis model in rat tibia, Pan et al. also performed immunohistochemical staining for the angiogenic factors CD31 and VEGFA and the osteogenic factors RUNX2 and OCN.^17^ They reported comparable levels of these angiogenic and osteogenic markers in the callus of their allogeneic group and autologous group that were significantly higher than in the cell-free group, indicating that autologous and allogeneic MSC promoted angiogenesis and osteogenesis.

Although most studies report broadly comparable outcomes with autologous and allogeneic MSC, one study suggests a measurable advantage for autologous cells.^32^ In a humanized mouse model, Rapp et al. created femoral defects and treated the defects with collagen gel containing autologous or allogeneic BM-MSC, then evaluated bone healing after 5 weeks using µCT and histomorphometry analyses.^32^ They found less bone formation with allogeneic BM-MSC (16 ± 4 % BV/TV) than with autologous BM-MSC (36 ± 6.5 % BV/TV). They also observed reduced expression of the primary osteogenic transcription factor Runx2 and impaired angiogenesis in terms of expression of CD31. Based on these findings, they concluded that autologous BM-MSC are superior to allogeneic cells for bone regenerative applications, and the mild early local immune response to allogeneic MSC might have contributed to the inferior bone regenerative capacity observed for the allogeneic MSC. While not included in the present systematic review, an ovine study by Coathup et al. is noteworthy with regards to superiority of autologous MSC.^86^ Using fibrin glue to deliver BM-MSC onto hydroxyapatite-coated ingrowth collars, the authors compared autologous BM-MSC groups with a group receiving 1×10^7^ allogeneic BM-MSC. In contrast to the autologous groups, the allogeneic MSC group demonstrated no extracortical bone formation and no bone/implant contact on histology, indicating inferior osteointegration when allogeneic cells were used in this setting.^86^

The included phase II trial^34^ provides the strongest direct clinical evidence in this review and aligns with the broader preclinical conclusion that allogeneic MSC use is feasible and safe in bone regeneration settings. The trial’s manufacturing strategy—expansion and osteoblastic pre-differentiation of AT-MSC before seeding into a tricalcium phosphate biomaterial—demonstrates a realistic translational pathway, including use of a master cell bank for the allogeneic product and a shorter procedural pathway by avoiding the autologous tissue-harvesting step. Clinically, healing was achieved in 83% of treated cases and was comparable to outcomes from a historical cohort treated with a fixed-dose autologous BM-MSC strategy, while procedure-related adverse events were limited. These findings support the concept that allogeneic AT-MSC biografts may offer a scalable alternative to autologous approaches, while also highlighting the need for larger controlled trials to clarify comparative efficacy.

### 4.4. Impact of MSC source on therapeutic efficacy and clinical translation

Most of the included studies used bone marrow as the MSC source, whereas adipose tissue appeared less frequently, specifically in one dog model and the clinical phase II trial. BM-MSC remains the most widely used cell source in bone tissue engineering, largely because of their well-described osteogenic capacity and long history in preclinical defect models and early clinical translation. However, adipose-derived MSC (AT-MSC) have emerged as a practical alternative, primarily due to easier harvesting and higher initial cell yield, which can reduce donor-site morbidity and simplify manufacturing logistics. Across comparative studies and reviews, AT-MSC typically demonstrates faster expansion and robust availability from lipoaspirates, whereas BM-MSC harvesting (aspiration) is more invasive and may yield fewer MSC per unit tissue, especially in older or comorbid donors.^62,87,88^ These pragmatic differences are frequently highlighted as reasons AT-MSC may be attractive for scalable, standardized products, including allogeneic strategies.^87,89^ In the preclinical studies in small and large animals which are included in this review, bone regeneration was evident when cells are combined with suitable scaffolds. Pointing in the same direction, the preclinical study using AT-MSC on a coral scaffold in a canine model achieved bone regeneration and defect bridging without persistent immune activation.

From an immunological and translational perspective, both BM-MSC and AT-MSC exhibit clinically relevant immunomodulatory properties, but they are not necessarily identical. Comparative studies show both sources can suppress immune effector functions, including T-cell and NK-cell responses, though the relative strength and mediator profiles (cytokines, soluble receptors, adhesion/migration factors) can differ across assays and inflammatory contexts.^88,90–92^ These differences may be particularly relevant for allogeneic applications, where persistence, immune recognition, and paracrine signaling are influenced by host immune environment. Despite the logistical advantages of AT-MSC, the literature suggests that osteogenic performance of AT-MSC is context-dependent, sometimes showing equal osteogenic differentiation capacity, but other times less capacity compared to BM-MSC, particularly in early time points and in certain scaffold environments.^58,93^ Importantly, donor-matched comparative work has also suggested that while AT-MSC can regenerate bone, the rate and/or magnitude of bone formation may be slower than BM-MSC in some bone-regeneration settings—an effect that could matter in large defects or when early mechanical stability is critical.^93^

The clinical efficacy of BM-MSC has been demonstrated, with good results, in long bone defects, in which autologous BM-MSC were used in combination with bioceramic.^94^ Another good example is the clinical study by Gjerde et al. in patients with severely atrophic mandibular ridges, where autologous BM-MSC combined with biphasic calcium phosphate resulted in significant new bone formation, sufficient volume for implant placement, and no serious adverse events.^6^ This was again confirmed in a randomized controlled clinical study comparing autologous BM-MSC combined with biphasic calcium phosphate to the gold standard bone autograft, achieving similar bone augmentation.^95^ The efficacy of AT-MSC has also been shown clinically.^96–98^ For instance, the phase II study included in this systematic review used expanded AT-MSC combined with a tricalcium phosphate biomaterial for atrophic nonunion, supporting feasibility and safety while underscoring the need for larger controlled comparisons and clearer potency markers across cell sources.^34^

#### 4.4.1. Need for osteogenic priming

One of the key challenges in the field of allogeneic MSC therapy for bone regenerative applications is ensuring consistent osteogenic differentiation. While MSC have the potential to differentiate into osteoblasts, the efficiency and consistency of this process *in vivo* remain variable. Factors such as scaffold material, local biological environment, and the presence of growth factors can influence MSC differentiation and subsequent bone regeneration.^9^

The included literature reflects two dominant cellular strategies in bone tissue engineering: implantation of non-differentiated MSC versus implantation of osteogenically primed MSC. When osteogenic priming was performed, it generally relied on the “classical” osteogenic cocktail: dexamethasone, ascorbate (commonly as ascorbic acid or ascorbic acid-2-phosphate), and β-glycerophosphate, added to a basal medium such as DMEM or DMEM/F12 (typically supplemented with serum and antibiotics) during cell culture before *in vivo* implantation or by incorporating growth factors, typically bone morphogenetic proteins (BMPs) with the *in vivo* implantation.

A rationale for using non-differentiated MSC is to preserve broad “MSC-like” behavior, including trophic and immunomodulatory signaling, while limiting *ex vivo* manipulation. In contrast, osteogenic induction aims to commit cells toward an osteoblast lineage before implantation, potentially improving early mineralization and reducing biological variability in demanding defects. In the included studies, osteogenic priming was used in the studies using AT-MSC, the preclinical study in canine cranial model and the human clinical trial, whereas it was not the same case for BM-MSC, with only 4 out of 11 studies using osteogenically primed cells. In the studies included in this systematic review, osteogenic priming of the MSC was applied to the cells during *in vitro* expansion in most of the studies, with a few studies priming the cells after seeding on to the scaffold before *in vivo* implantation. Generally, osteogenic priming of the cells was performed for periods of 1-3 weeks before *in vivo* implantation.

The need for osteogenic priming for AT-MSC, unlike BM-MSC, can be due to the inherent osteogenic capacity of BM-MSC, which does not need priming before *vivo* implantation. In fact, it has been previously reported that BM-MSC did not necessarily need osteogenic stimulation before subcutaneous *in vivo* implantation to form ectopic bone-like tissue, whereas osteogenic stimulation of AT-MSC was important for ectopic mineralized tissue formation *in vivo*.^99^ A widely cited synthesis notes that while both BM-MSC and AT-MSC can form mineralized tissue *in vitro* and contribute to bone formation *in vivo*, BM-MSC may display more consistent osteogenic commitment under “standard” conditions, whereas AT-MSC sometimes benefit more from osteogenic priming or stronger osteoinductive cues.^89^

A noteworthy pattern within the clinical application of AT-MSC for bone regenerative applications is the consistent incorporation of osteogenic priming.^34,96–98^ This may reflect an investigator preference to “standardize” osteogenic behavior when using adipose-derived cells, although direct head-to-head trials comparing primed vs non-primed MSC within the same model were not a primary focus of the comparative autologous/allogeneic studies included here.

Although the following approaches fall outside our inclusion criteria, they are relevant to the discussion because many studies aim to enhance MSC osteogenic efficacy through “cell modification.” A prominent strategy is genetic engineering to overexpress osteogenic/angiogenic factors (e.g., BMPs, VEGF, RUNX2), which accelerated defect bridging and increased bone volume in preclinical models compared with unmodified MSC.^100–103^ However, most gene-modified products rely on viral vectors, raising translational concerns (insertional mutagenesis, prolonged/ectopic expression) and adding regulatory complexity, particularly for standardized allogeneic products.^102,103^ Beyond gene transfer, osteogenesis can also be improved via miRNA modulation, hypoxic/inflammatory preconditioning to boost survival and homing, and bioengineering strategies (functionalized surfaces, nanoparticles, and advanced 3D-printed scaffolds/hydrogels) that promote retention, vascularization, and defect-specific regeneration.^100,101,103–108^

Despite these promising advances, reviews consistently stress that there is still limited *in vivo* data directly comparing modified versus unmodified MSC in bone defect models, and virtually no head-to-head clinical trials.^100,101,103^ Many of the most potent approaches (e.g. viral gene transfer, inorganic nanoparticles) raise concerns regarding long-term safety, immunogenicity, off-target biodistribution and GMP-grade manufacturing.^101–103^ For allogeneic, standardized constructs intended for bone therapeutic applications, a realistic translational pathway may therefore prioritize simpler, scalable modifications: short-lived gene delivery (non-integrating vectors or mRNA), hypoxic or pharmacological preconditioning, and scaffold-bound osteoinductive cues that avoid permanent cell modification.^101–103,107–109^ Integrating such strategies into future preclinical models of bone reconstruction—and ultimately into clinical trials—could reduce the variability we observed with unmodified allogeneic MSC and help move the field towards more consistent and predictable bone regeneration outcomes.^100–103,109^

#### 4.4.2. Influence of cell dose on bone formation

Cell dose varied widely across studies and was strongly linked to animal and defect size, and delivery approach. At the lower end, Pan et al. injected 5×10^5^ cells locally into a rat distraction model in rats and found comparable bone formation between autologous and allogeneic BM-MSC groups by radiography and µCT. At the higher end, Berner et al. implanted 35×10^6^ osteogenically induced MSC in a large ovine segmental defect model and similarly observed no significant differences between autologous and allogeneic groups. Importantly, most studies used single-dose designs, limiting inference regarding dose response relationships. The clinical trial is notable because it explicitly scaled dose to defect location/size (6×10^6^-56×10^6^ AT-MSC), showing high healing rates and acceptable safety in both autologous and allogeneic settings. Collectively, the included evidence supports feasibility across a broad dosing range, but it does not yet define a minimum effective dose or demonstrate a consistent “more cells = more bone” relationship under standardized conditions.

Overall, current evidence supports the view that BM-MSC are often a strong “default” choice for osteogenesis, while AT-MSC offer major logistical advantages (harvesting burden and scalable yield) and can achieve clinically meaningful bone repair when paired with appropriate scaffolds and/or osteoinductive conditioning. Accordingly, one source may not be considered universally superior, but the cell source should be selected in conjunction with a patient’s condition, scaffold selection, need for priming, defect biomechanics, and manufacturing constraints, especially when aiming for standardized allogeneic products.^87,89,93^

### 4.5. Summary of the meta-analysis

Our meta-analysis suggests that allogeneic MSC combined with osteoconductive scaffolds can support clinically relevant bone regeneration, but with substantial heterogeneity in effect sizes between studies. After a systematic literature search for this study, we excluded a large number of studies in which modified versions of MSC were used. We did this to obtain the least heterogeneous material possible and thereby enable optimal comparison between studies. Nevertheless, it is necessary to discuss this area to gain a broader understanding of the field and to more easily relate it to the reviewed literature. This has driven interest in *functionalized* or modified MSC products that aim to make osteogenesis more robust and predictable. Recent reviews specifically focused on MSC modification for bone defect repair highlight that engineering the cells themselves, rather than only optimizing scaffold or delivery route, may be crucial to overcome limitations such as poor *in vivo* survival, suboptimal homing, and inconsistent osteogenic differentiation.^100,101^

### 4.6. Limitations and future directions

Methodological heterogeneity was a major limitation across the included studies, with substantial variation in animal models, defect sites, scaffolds, MSC source and processing, dosing, delivery route, follow-up duration, and outcome measures. This makes it difficult to draw definitive conclusions regarding the superiority or equivalence of allogeneic versus autologous MSC in bone repair.^110^ Reporting quality was inconsistent: several studies relied on subjective scoring or did not provide quantitative bone volume data, and key details (e.g., animal numbers, controls, and cell preparation) were often incomplete. As a result, only a subset of studies could be included in the meta-analysis, numerical values sometimes had to be estimated from figures, and the overall evidence base predominantly preclinical has limited generalizability to humans. Although the funnel plot did not suggest marked publication bias, this cannot be ruled out given the small number of studies.

A further limitation of the pooled analysis is that it combines data across species with substantially different bone physiology, healing capacity, and defect scale, ranging from rodents to large animals and a single clinical trial. Although the statistical heterogeneity across studies was low (I^2^ = 11.39%), this reflects consistency in standardized effect sizes rather than biological comparability across species, and the pooled estimate should therefore be interpreted as a directionally consistent summary rather than a single value that is generalizable across species or directly applicable to clinical practice. Future reviews, once a larger evidence base is available, should consider species-stratified or clinical-versus-preclinical analyses as separate comparisons.

An additional limitation relates to data extraction. For several included studies, quantitative outcome data were not reported numerically and were instead extracted from published graphs by manual measurement using a ruler. This manual approach introduces a greater degree of measurement uncertainty relative to studies reporting exact numerical values, which may affect the precision, though not necessarily the direction, of the pooled effect estimates. This should be considered when interpreting the magnitude, rather than the overall direction, of the meta-analytic findings.

Despite these constraints, this review has important strengths: it is, to our knowledge, the first head-to-head systematic review with meta-analysis comparing autologous versus allogeneic MSC for bone regeneration. Restricting inclusion to direct comparisons improved internal validity, and quantitative synthesis was performed where data permitted.

The use of allogeneic MSC represents a promising frontier in regenerative medicine, particularly for bone reconstruction. Their immediate availability and scalable production offer distinct advantages over autologous sources, especially in urgent clinical scenarios. For clinical translation, future studies should standardize protocols for MSC isolation/culture and harmonize outcome assessment, especially bone volume, mechanical strength, and immune responses with consistent reporting of controls and methodological details. To fully realize their therapeutic potential, future studies on allogeneic MSC should also focus on optimal donor selection and identification of optimal biomaterial scaffolds that support osteogenesis and mechanical integration. Additional work should also address systemic delivery strategies, where inefficient homing remains a bottleneck. Most current evidence is derived from preclinical animal studies, and while these are valuable for mechanistic insights, they do not always translate directly to human outcomes due to interspecies differences in immune response and bone biology.^111^ Larger, well-designed long-term human trials of allogeneic MSC are needed to clarify long-term safety and comparative efficacy, alongside studies optimizing standardized protocols for human applications. Finally, cost-effectiveness evaluations and emerging technologies may support broader adoption of allogeneic MSC for therapeutic applications. A multidisciplinary approach integrating biomaterials science, immunology, and clinical expertise will be key to translating allogeneic MSC therapies into routine clinical practice.

## 5. Conclusion

Based on the reviewed data we can conclude with the following:

• Allogeneic MSC shows regenerative outcomes broadly comparable to autologous MSC in preclinical models, particularly when combined with osteoconductive or bioactive scaffolds, although evidence from adequately powered clinical trials remains limited.
• Minor advantages in osteogenic or angiogenic markers were reported for autologous MSC in some studies, but not consistently across the included literature.
• Reported immune reactions to allogeneic MSC were generally mild across studies using heterogeneous assessment methods and did not appear to impair bone healing; however, these findings should be interpreted with caution given differences in the outcome measures used.
• Allogeneic MSC offer practical advantages, including immediate availability, scalable production, and avoidance of donor-site morbidity.
• The current evidence base is limited by substantial methodological heterogeneity across species, scaffold types, cell sources, and outcome measures, and by reliance predominantly on preclinical models, with only a single clinical trial included.
• Taken together, these findings are encouraging but remain preliminary. Confirmation through adequately powered, well-designed randomized clinical trials are required before allogeneic MSC can be considered for routine clinical translation in bone regeneration.

## Supplemental material

Supplemental Material - Allogeneic stem cell strategies in bone regeneration: A systematic review and meta-analysis Supplemental Material for Allogeneic stem cell strategies in bone regeneration: A systematic review and meta-analysis by Paul Åsmund Vågen, Lado Lako Loro, Samih Mohamed-Ahmed, Stein Atle Lie, Mohammed Ahmed Yassin, Cecilie Gjerde in Journal of Tissue Engineering
