# Supplementary material for: Allogeneic stem cell strategies in bone regeneration: A systematic review and meta-analysis
Source: J Tissue Eng. 2026 Sep 17;17:20417314261488564. doi: 10.1177/20417314261488564 (PMC13724909; doi:10.1177/20417314261488564)
Supplement: Supplementary file 1 [file sj-pdf-1-tej-10.1177_20417314261488564.pdf]

Supplementary

Search strategy in Medline and Embase:

(((Bone or bones or osteo*) adj3 (regenerat* or regrowth* or defect* or repair* or heal*
or form*)) or osteosis).ti,ab,kf.

((Allogenic or allogeneic or allo*) adj5 (ASC or ASCs or MSC or MSCs or BMSC or BMSCs or multipotent or multi-potent or mesenchymal or progenitor cell* or stromal cell* or
stem cell*)).ti,ab,kf.

((autologous or autogenic or autogeneic or auto*) adj5 (ASC or ASCs or MSC or MSCs or BMSC or BMSCs or multipotent or multi-potent or mesenchymal or progenitor cell*
or stromal cell* or stem cell*)).ti,ab,kf.

SYRCLE Risk of bias table:

Table 1: SYRCLE risk of bias assessment

Studie	Randomization of animal allocation	Allocation concealment	Blinding of personnel	Blinding of outcome assessor	Complete outcome data	Selective reporting	Other bias
Arinzeh et al. (2003)	Yes	No	No	Yes	Yes	Unclear	Possible immune response
Kok et al. (2003)	Yes	Yes	No	Yes	Yes	Yes	No major issues
Guo et al. (2009)	Yes	Yes	No	Yes	Yes	Yes	No major issues
Kang et al. (2013)	Yes	Yes	No	Yes	Yes	Yes	No major issues
Liu et al. (2013)	Yes	No	No	Yes	Yes	Unclear	Possible immune response
Udhiya et al. (2013)	Yes	Yes	No	Yes	Yes	Yes	No major issues
Berner et al. (2013)	Yes	Yes	No	Yes	Yes	Yes	No major issues
Wu et al. (2016)	Yes	No	No	Yes	Yes	Unclear	Possible immune response

Maiti et al. (2018)	Yes	Yes	No	Yes	Yes	Yes	No major issues
Rapp et al. (2018)	Yes	Unclear	No	Yes	Yes	Unclear	Possible immune response
Pan et al. (2021)	Yes	Yes	No	Yes	Yes	Yes	No major issues
Chu et al. (2021)	Yes	No	No	Yes	Yes	Unclear	No major issues
Delclos-Orozco et al. (2024)	Yes	No	No	Yes	Yes	Unclear	No major issues

The SYRCLE risk of bias assessment indicated an overall moderate risk of bias among the included studies. Although randomization, outcome assessor blinding, and completeness of outcome data were generally well reported, most studies lacked personnel blinding, and allocation concealment and selective reporting were often unclear. In addition, possible immune response was identified as a potential source of other bias in several studies.
